# Maximal sfermion flavour violation in super-GUTs

**DOI:** 10.1140/epjc/s10052-016-4398-9

**Published:** 2016-10-20

**Authors:** John Ellis, Keith A. Olive, L. Velasco-Sevilla

**Affiliations:** 1 Theoretical Particle Physics and Cosmology Group, Department of Physics, King’s College London, London, WC2R 2LS UK; 2 Theoretical Physics Department, CERN, 1211 23 Geneva, Switzerland; 3 William I. Fine Theoretical Physics Institute, School of Physics and Astronomy, University of Minnesota, Minneapolis, MN 55455 USA; 4 Department of Physics and Technology, University of Bergen, PO Box 7803, 5020 Bergen, Norway

## Abstract

We consider supersymmetric grand unified theories with soft supersymmetry-breaking scalar masses $m_0$ specified above the GUT scale (super-GUTs) and patterns of Yukawa couplings motivated by upper limits on flavour-changing interactions beyond the Standard Model. If the scalar masses are smaller than the gaugino masses $m_{1/2}$, as is expected in no-scale models, the dominant effects of renormalisation between the input scale and the GUT scale are generally expected to be those due to the gauge couplings, which are proportional to $m_{1/2}$ and generation independent. In this case, the input scalar masses $m_0$ may violate flavour maximally, a scenario we call MaxSFV, and there is no supersymmetric flavour problem. We illustrate this possibility within various specific super-GUT scenarios that are deformations of no-scale gravity.

## Introduction

Ever since the earliest days of supersymmetric model-building, it has been emphasised that data on flavour-changing processes suggest the existence of a ‘super-GIM’ mechanism to ensure that the effective electroweak-scale slepton and squark mass matrices are almost diagonal with small generational mixing and eigenvalues that are almost degenerate [1]. This constraint on supersymmetric model-building has subsequently been dubbed the ‘supersymmetric flavour problem’. Soon after [1], it was recognised that one possible scenario for solving this ‘problem’ in the squark sector would be to postulate that all supersymmetric flavour violation is proportional to the Cabibbo–Kobayashi–Maskawa (CKM) mixing between quarks [2], a scenario that has come to be known as minimal flavour violation (MFV). This approach left open the question how MFV came to be, one possible answer being provided by gaugino mediation of supersymmetry breaking [3–5].

A suitable framework for studying the supersymmetric flavour problem is provided by a supersymmetric GUT such as SU(5) [6, 7] in which the soft supersymmetry-breaking scalar masses $m_0$, the trilinear soft supersymmetry-breaking parameters $A_0$ and the gaugino masses $m_{1/2}$ are input at the GUT scale $M_\mathrm{GUT} \simeq 10^{16}$ GeV. Upper limits on the deviations from Standard Model predictions for flavour-changing processes motivate the hypothesis that the $m_0$ parameters for chiral supermultiplets with the same gauge quantum numbers are identical at this input scale [2], and the GUT symmetry requires them to be identical for all the sparticles in the same GUT multiplet. Thus, in SU(5) all the $\varvec{\bar{5}}$ sfermions would have a common $m_0$, and all the $\mathbf {10}$ sfermions would have another (potentially different) common $m_0$. It is often assumed, with no clear phenomenological motivation apart from simplicity and possible embedding in a larger supersymmetric GUT such as SO(10), that these two $m_0$ parameters are identical at the GUT scale, a scenario called the constrained minimal supersymmetric extension of the standard model or CMSSM [8–25].

The question remains, however, what might be the origin of any such universality in the $m_0$ parameters. This would occur in minimal supergravity models with trivial, flat Kähler metrics [26], but would not happen in more general supergravity models [27], as discussed recently in the context of compactified M-theory [28]. One interesting exception is no-scale supergravity [30, 31], in which the input soft supersymmetry-breaking scalar masses $m_0$ vanish at the input scale. In this case, the electroweak-scale soft supersymmetry-breaking scalar masses are all generated by gauge interactions, and hence are identical for different sparticles with the same gauge quantum numbers, in a manner reminiscent of gauge-mediated supersymmetry-breaking models [32]. The phenomenological constraints on sparticle masses exclude models with no-scale boundary conditions at the supersymmetric GUT scale [33], but no-scale boundary conditions at higher input scales may be acceptable [33–35].

The principal purpose of this paper is to study the constraints on the flavour structure of the soft supersymmetry-breaking scalar masses for models that are similar to such no-scale models, with $m_0 \ll m_{1/2}$ at some input scale $M_\mathrm{in} > M_\mathrm{GUT}$, scenarios we call super-GUTs. One may regard such scenarios as deformations of the simple no-scale framework, as might occur in realistic string models via higher-order corrections to the (over-simplistic?) no-scale Kähler potential, cf, the studies in [28]. Intuitively, it is clear that the constraints on the non-universalities between the diagonal $m_0$ parameters and on the ratios of off-diagonal to diagonal entries in the soft supersymmetry-breaking scalar mass matrix must become progressively weaker as the no-scale limit: $m_0 \rightarrow 0$ is approached.

Indeed, close to this no-scale limit a completely anarchic $m_0$ matrix is allowed. In this sense, we consider possible anarchic structures which we term as maximal flavour violation (MaxSFV). Thus, there is no ‘supersymmetric flavour problem’ for super-GUTs with input boundary conditions at some scale $M_\mathrm{in} > M_\mathrm{GUT}$ that are small deformations of the idealised no-scale limit. A primary objective of this paper is to quantify this statement within illustrative super-GUT scenarios.

The effects of flavour-violating sfermion mass parameters on hadronic and leptonic flavour observables, as well as their correlations, have been studied previously in the context of Grand Unified Theories. These studies typically assume the mass-insertion approximation without a complete top-down running of the soft supersymmetry-breaking parameters (see, e.g., [29]). We do not strive to study the generalities of such correlations, instead we consider a specific set up where we establish limits on the maximal values of the off-diagonal entries in the $m_0$ matrix using a complete running of soft supersymmetry-breaking parameters. Also, we constrain the parameter space via EW observables as well as flavour-violating effects. To our knowledge, this is the first complete and realistic study that takes into account running from a scale above the unification scale.

The organisation of this paper is as follows. We begin in Sect. 2 by setting up our super-GUT model framework [33, 34, 36–41], focusing in particular on its implementation in no-scale supergravity [34]. We use weak-scale measurements to specify the gauge and Yukawa couplings, whereas the soft supersymmetry-breaking scalar masses, trilinear and bilinear terms are specified at the input scale, $M_{ in }$. The matching conditions at $M_\mathrm{GUT}$ are discussed in Sect. 2.3. We then specify in Sect. 2.4 the illustrative flavour-mixing models that we choose for further study. In Sect. 3 we analyse the case of pure no-scale boundary conditions, in which all soft supersymmetry-breaking scalar masses, trilinear and bilinear terms are set to zero at $M_{ in }$. We display the running of these parameters as well as the Yukawa couplings between the input and weak scales for our representative flavour-mixing scenarios. Then, in Sect. 4, we analyse super-GUT scenarios in which $M_{ in }> M_\mathrm{GUT}$, studying the upper bounds on non-universality in $m_0/m_{1/2}$ that are permitted by the experimental upper limits on flavour-changing interactions as functions of $M_{ in }$ in our illustrative flavour-mixing scenarios. Finally, Sect. 5 summarises our conclusions.

## Model framework

### No-scale SUGRA model

We first consider a low-energy effective theory that is based on an $N = 1$ supergravity model with the simplest no-scale structure [30, 31] defined by a Kähler potential

1 $$\begin{aligned} K \; = \; - 3 \mathrm{ln} \left( T + T^\dagger - \sum _i |\Phi _i|^2/3\right) \end{aligned}$$

where *T* is a modulus field and $\Phi$ represents matter fields present in the theory. The no-scale form (1) for *K* ensures that all soft supersymmetry-breaking scalar masses and bi- and trilinear terms vanish at some input universality scale, $M_{ in }$. However, we recall that non-zero gaugino masses arise independently from a non-trivial gauge kinetic function $f_{\alpha \beta }$ in the effective supergravity theory. It is well known that vanishing soft masses at the GUT scale $M_\mathrm{GUT}$ in a theory with universal gaugino masses are in general phenomenologically disastrous [33]. However, this problem may be circumvented if the input universality scale is between the GUT scale and the Planck scale [33–35].

A Kähler potential of the form (1) arises in generic manifold compactifications of string theory, in which *T* is identified as the manifold volume modulus. In such a scenario, the $\Phi _i$ are identified as untwisted matter fields. In general there would, in addition, be twisted matter fields $\varphi _a$ described by additional terms in the Kähler potential of the form

2 $$\begin{aligned} \Delta K \; = \; \sum _a \frac{|\varphi _a|^2}{(T + T^\dagger )^{n_a}} \, , \end{aligned}$$

where the parameters $n_a$ are model-dependent modular weights. These give rise to $n_a$-dependent soft supersymmetry-breaking terms whose magnitudes and flavour structure are also model-dependent and violate the MFV assumption, in general. Here, we assume that the MSSM matter fields are assigned to the untwisted sector.

The renormalisation of MSSM parameters at scales above $M_\mathrm{GUT}$ requires the inclusion of new particles and parameters in addition to those in the generic MSSM, including GUT-scale Higgses, their self-couplings and couplings to matter. For simplicity, we assume here minimal SU(5), in which one introduces a single SU(5) adjoint Higgs multiplet $\hat{\Sigma }(\mathbf {24})$, and the two Higgs doublets of the MSSM, $\hat{H}_d$ and $\hat{H}_u$, are extended to five-dimensional SU(5) representations $\hat{\mathcal {H}}_1(\mathbf {\overline{5}})$ and $\hat{\mathcal {H}}_2(\mathbf {5})$, respectively. The minimal renormalisable superpotential for this model is [40, 42, 43]

3 $$\begin{aligned} W_5= & {} \mu _\Sigma {{\mathrm{Tr}}}\hat{\Sigma }^2 + \frac{1}{6}\lambda '{{\mathrm{Tr}}}\hat{\Sigma }^3 + \mu _H \hat{\mathcal {H}}_{1\alpha } \hat{\mathcal {H}}_2^{\alpha } + \lambda \hat{\mathcal {H}}_{1\alpha }\hat{\Sigma }^{\alpha }_{\beta } \hat{\mathcal {H}}_2^{\beta } \nonumber \\&+\frac{1}{4}({\varvec{ h}_{10}})_{ij} \epsilon _{\alpha \beta \gamma \delta \zeta } \hat{\psi }^{\alpha \beta }_i \hat{\psi }^{\gamma \delta }_j \hat{\mathcal {H}}_2^{\zeta } +\sqrt{2}({\varvec{ h}_{\overline{5}}})_{ij} \hat{\psi }^{\alpha \beta }_i \hat{\phi }_{j\alpha } \hat{\mathcal {H}}_{1\beta } , \nonumber \\ \end{aligned}$$

where Greek letters denote SU(5) indices, $i,j=1,2,3$ are generation indices and $\epsilon$ is the totally antisymmetric tensor with $\epsilon _{12345}=1$. The $\hat{D}^c_i$ and $\hat{L}_i$ superfields of the MSSM reside in the $\mathbf {\overline{5}}$ representations, $\hat{\phi }_i$, while the $\hat{Q}_i,\ \hat{U}^c_i$ and $\hat{E}^c_i$ superfields are in the $\mathbf {10}$ representations, $\hat{\psi }_i$. The new dimensional parameters $\mu _H$ and $\mu _\Sigma$ are of $\mathcal {O}(M_{\mathrm{GUT}})$. The soft supersymmetry breaking part of the Lagrangian involving scalar components of chiral superfields can then be written as

4 $$\begin{aligned}&\mathcal {L}_{soft}(\psi ,\phi ) \ni -m^2_{\bar{5}} |\phi |^2 - m^2_{10} \mathrm {Tr} \left[ \psi ^\dagger \psi \right] \nonumber \\&\quad + \left[ b_\Sigma {{\mathrm{Tr}}}\hat{\Sigma }^2 +\frac{1}{6} a' {{\mathrm{Tr}}}\hat{\Sigma }^3 + b_H \hat{\mathcal {H}}_{1\alpha } \hat{\mathcal {H}}_2^{\alpha } + a \hat{\mathcal {H}}_{1\alpha }\hat{\Sigma }^{\alpha }_{\beta } \hat{\mathcal {H}}_2^{\beta } \right] \nonumber \\&\quad + \left[ \frac{1}{4} \ a_{10}\ \epsilon _{ijklm} \psi ^{ij}\psi ^{kl} \ {\hat{\mathcal {H}}}_2^m + \sqrt{2} \ a_{\bar{5}} \ \psi ^{ij} \phi _i \ {\hat{\mathcal {H}}}_{1j} + H.c. \right] , \nonumber \\ \end{aligned}$$

where the soft parameters $\{a, b \}$ are assumed to be of the same order as $m^2_{\bar{5}}$ and $m^2_{10}$, and hence of $\mathcal {O}(M_\mathrm{weak})$.

### Boundary conditions at $M_{ in }$

The no-scale structure (1) requires that all supersymmetry-breaking soft masses and bi- and trilinear terms for the fields $\Phi _i$ vanish at $M_{ in }$, so that

5 $$\begin{aligned} m_0=B_0=A_0=0, \end{aligned}$$

where the bilinear couplings $b_\Sigma$ and $b_H$ in (4) are related to the corresponding superpotential terms by

6 $$\begin{aligned} b_\Sigma \; \equiv \; B_{\Sigma } \mu _{\Sigma },\quad b_H \; \equiv \; B_{H} \mu _{H}. \end{aligned}$$

and the trilinear couplings $a', a, a_{10}$ and $a_{\bar{5}}$ in (4) are related to the corresponding Yukawa couplings by

7 $$\begin{aligned}&a' \; \equiv \; A' \lambda ',\quad a \; \equiv \; A \lambda ,\quad (a_{10})_{ij}\; \equiv \; A_{10 ij} (h_{10})_{ij},\quad \nonumber \\&\quad (a_{\bar{5}})_{ij} \; \equiv \; A_{5 ij} (h_{\bar{5}})_{ij} , \end{aligned}$$

**Table 1:** Values of the fermion masses, in GeV, as appear in current edition of the PDG review [47]. The quoted quark mass values at $M_Z$ were obtained with the program RunDec [48]. The values of the charged-lepton masses were taken from [49]

	Mass values (GeV)	$$\begin{array}{c} m^{\overline{MS}}_f(M_Z) \text {(GeV)}\end{array}$$
${m}_t$	$173.21\pm 0.51\pm 0.71$	$171.46 \pm 0.96$
$m_b$	$4.18\pm 0.03$	$2.85 \pm 0.04$
$m_c$	$1.275\pm 0.025$	$0.63 \pm 0.025$
$m_s$	$0.095\pm 0.005$	$0.059 \pm 0.0033$
$m_d$	$4.8^{+0.5}_{-0.3}\times 10^{-3}$	$0.0028 \pm 0.0004$
$m_u$	$2.3^{+0.7}_{-0.5}\times 10^{-3}$	$0.0013 \pm 0.0005$
$m_e$	$(0.51 \pm (1.1\times 10^{-8} ) ) \times 10^{-3}$	$(0.49\pm (4.2\times 10^{-8}))\times 10^{-3}$
$m_\mu$	$(105.66 \pm (3.5 \times 10^{-6}))\times 10^{-3}$	$(102.72\pm (9.2 \times 10^{-6}))\times 10^{-3}$
$m_\tau$	$1.78\pm (1.2\times 10^{-4})$	$1.75\pm (2\times 10^{-4})$

where no summation over repeated indices is implied. Having specified the boundary conditions on scalar masses as well as setting $A_0 = B_0 = 0$, we no longer have the freedom of choosing $\tan \beta$ as a free parameter. Instead, the minimisation of the Higgs potential provides the solutions for both the MSSM Higgs mixing parameter $\mu$ and $\tan \beta$ [44]. Thus the theory is defined by 4 parameters:

8 $$\begin{aligned} m_{1/2},\ M_\mathrm{in},\ \lambda ,\ \lambda ', \end{aligned}$$

where we will denote the gaugino mass above the GUT scale by $M_5$. In addition, the sign of the MSSM $\mu$ parameter must also be specified.^1^

### Boundary conditions at $M_{ GUT }$

In the previous subsection, we specified the boundary conditions on the soft supersymmetry-breaking parameters at the input universality scale $M_{ in }$. Using the GUT RGEs, these are run down to $M_{\mathrm{GUT}}$ where they must be matched with their MSSM equivalents. At the GUT scale, we have

9 $$\begin{aligned} \begin{array}{ll} M_i=M_5, \\ m^2_{D}=m^2_{L}=m^2_{\bar{5}}, \quad a_t= {4} a_{10},\\ m^2_{Q}=m^2_{U}=m^2_{E}=m^2_{10}, \quad a_b=a_\tau =a_{\bar{5}}/{{\sqrt{2}}} ,\\ m^2_{H_d}=m^2_{\mathcal {H}_1}, \quad m^2_{H_u}=m^2_{\mathcal {H}_2}.\\ \end{array} \end{aligned}$$

We treat the gauge and Yukawa couplings differently, inputting their values at the electroweak scale and matching to their SU(5) counterparts at $M_{ GUT }$. The minimal SU(5) relations

10 $$\begin{aligned} h_E(M_{ GUT })= {h_D(M_{ GUT })}^T, \end{aligned}$$

between the charged-lepton and for the down-type Yukawa couplings are unrealistic since they do not produce the right values of lepton masses at $M_{ EW }$, except possibly for the third generation. We assume here that at $M_{ GUT }$

11 $$\begin{aligned} h_D(M_{ GUT })= {h_{\bar{5}}(M_{ GUT })}/\sqrt{2}, \end{aligned}$$

but we do not match $h_E$ to $h_5$ at $M_{ GUT }$. Instead we use the values that $h_E$ should have at $M_{ GUT }$ in order to produce the observed lepton masses. One way to justify this assumption would be to allow the lepton sector to have additional, non-renormalisable couplings besides the minimal renormalisable $\bar{5}$ couplings [46], so at $M_{ GUT }$ one has

12 $$\begin{aligned} h_E(M_{ GUT })= {h_{\bar{5}} (M_{ GUT })}^T/\sqrt{2} + \ \text {other interactions}, \end{aligned}$$

where these other interactions are too small to be important for the quark sector. Thus for the gauge and Yukawa couplings, we determine their SU(5) counterparts as

13 $$\begin{aligned}&g_i=g_5 \nonumber , \\&h_t=4 h_{10}, \nonumber \\&(h_D+ h_E) _{33}/2= {h_{\bar{5}}}_{33}/\sqrt{2}, \nonumber \\&{h_D(M_{ GUT })}_{ij}= {h_{\bar{5}}(M_{ GUT })}_{ij}/\sqrt{2}, \forall \ \{i,j\} \ \text {except}\ \{i,j\}={33},\nonumber \\ \end{aligned}$$

The corresponding experimental inputs for the Yukawa couplings at the weak scale are shown in Table 1.

We use for our renormalisation-group calculations the program SSARD [50], which computes the sparticle spectrum on the basis of 2-loop RGE evolution for the MSSM and 1-loop evolution for minimal SU(5). We define $M_{\mathrm{GUT}}$ as the scale where $g_1=g_2$, so that $M_{\mathrm{GUT}}\simeq 10^{16}$ GeV, with its exact value depending on the values of other parameters. The value of $g_3$ at $M_{\mathrm{GUT}}$ is within the threshold uncertainties in the GUT matching conditions.

### Non-zero off-diagonal Yukawa couplings

It is well known that renormalisation interrelates the soft masses-squared, trilinear and Yukawa couplings. In particular, a non-zero diagonal soft mass-squared term, the Kähler potential, the F terms and the Yukawa couplings could be seeds for non-zero off-diagonal soft masses-squared and trilinear terms. Alternatively, even if the Yukawa couplings were flavour-diagonal, there would be non-diagonal soft masses-squared and trilinear terms if the Kähler potential [28] or the F terms were flavour non-diagonal.

However, in the case of no-scale supergravity boundary conditions at $M_{ in }$ there is no source of non-zero trilinear or soft masses-squared, apart from the running induced by the renormalisation-group $\beta$ functions. However, off-diagonal Yukawa couplings at $M_{ in }$ would generate, via renormalisation-group running, off-diagonal soft masses-squared and trilinear terms. The ultimate goal of our study is to quantify how large these parameters could be near $M_{ in }$ before they become problematic at $M_{ EW }$.

In order to understand the effects of the Yukawa couplings on the evolution of the soft supersymmetry-breaking parameters, we initialise our study by enforcing conditions at $M_{ EW }$ that reproduce the CKM matrix. In particular, we take the values of the quark masses at $M_{ EW }$ to be those at $M_Z$ (see the second column of Table 1), and use $h_D(M_Z)=\sqrt{2}\, m_D(M_Z)/v/\cos \beta$ and $h_U(M_Z)=\sqrt{2}\, m_U(M_Z)/v/\sin \beta$. The diagonalisation of the Yukawa couplings is defined by

14 $$\begin{aligned} h_D=V^D_R\hat{h}_D V^{D\dagger }_L,\quad h_E=V^E_R\hat{h}_E V^{E\dagger }_L, \end{aligned}$$

where $\hat{h}_D$ and $\hat{h}_E$ are diagonal matrices, and the unitary matrices $V_{L, R}^{D, E}$ are such that the CKM matrix is $V^{U\dagger }_L V^D_L$.

Since the structure of the Yukawa couplings cannot be determined in a model-independent way, we adopt the minimal assumption that the CKM matrix is the only source of flavour violation in the Yukawa couplings in the *D* sector, and study the differences induced by different assumptions for the *E* sector. Thus, we are assuming that $h_U$ is diagonal at $M_{ EW }$. Using this condition, 1-loop running does not generate off-diagonal terms, and the off-diagonal entries generated at $M_{ GUT }$ at the 2-loop level are negligibly small.

We remind the reader that, in contrast to the Standard Model, the MSSM observables are sensitive to right-handed currents, which have no Standard Model counterparts. Therefore, it is not possible to make predictions without additional assumptions^2^ on the Yukawa couplings, and hence the $V_R^{D, E}$ as well as the $V_L^{D, E}$.

We therefore consider the following illustrative Ansätze that illustrate the range of possibilities:

15 $$\begin{aligned} \mathrm{A1.}&V^{D*}_R=V^{D}_L=V_\mathrm{{CKM}}, \quad \quad \quad V^{E}_R=V^E_L=1, \end{aligned}$$

16 $$\begin{aligned} \mathrm{A2.}&V^D_L=V_\mathrm{{CKM}}, \quad V^D_R=1, \quad \ V^E_R=V^E_L=1, \end{aligned}$$

17 $$\begin{aligned} \mathrm{A3.}&V^D_R =V^{E}_L=1,\quad \quad \quad \quad \quad \ V^D_L=V^{E*}_R=V_\mathrm{{CKM}}, \end{aligned}$$

18 $$\begin{aligned} \mathrm{A4.}&V^{D*}_R=V^D_L=V_\mathrm{{CKM}}, \quad \quad \quad V^{E*}_R=V^E_L=V_\mathrm{{CKM}}.\nonumber \\ \end{aligned}$$

The Ansätze A3 and A4 are compatible with the minimal SU(5) conditions (10) and (14). However, we will focus later on Ansätze A1 and A2 because, as we shall see, Ansätze A3 and A4 give rise to unacceptably large flavour-violating processes in the lepton sector. We use examples A1 and A2 to illustrate the determination of the diagonal and off-diagonal soft masses-squared and trilinear terms that are generated below $M_{ in }$. These are constrained by flavour observables, and our goal is to determine how large the deviations from pure no-scale boundary conditions can be, before they induce flavour violations in contradiction with experiment.

## Running of parameters

In this section, we restrict our attention to pure no-scale boundary conditions, and assume the Ansatz A1 for the CKM mixing among fermions. As already mentioned, the boundary conditions for the soft supersymmetry-breaking parameters are fixed at $M_{ in }$, the gauge and Yukawa couplings are fixed at the weak scale, and all parameters are matched at $M_{\mathrm{GUT}}$ to allow for running above and below the GUT scale. Though the off-diagonal sfermion masses begin their RGE evolution with the no-scale boundary conditions, i.e., they vanish at $M_{ in }$, they contribute to low-energy flavour observables in a model-dependent way after renormalisation, as we now calculate.

To be concrete, we choose a limited set of benchmark points with different choices of $\lambda$, $\lambda '$ and $M_{ in }$ (all masses are expressed in GeV units). The four-dimensional parameter space of the super-GUT no-scale model was explored in [34]. It was found that unless $\lambda /\lambda ' < 0$ with $|\lambda | < |\lambda ' |$, both $m_{1/2}$ and $M_{ in }$ are pushed to relatively low values. However, the low values of $m_{1/2}$ are now in conflict with LHC searches for supersymmetric particles [52–54]. Therefore we restrict our analysis to an illustrative benchmark point **B** defined by

19 $$\begin{aligned}&\mathbf {B}: M_5=1500~\mathrm{GeV}, \quad M_{ in }=1\times 10^{18}~\mathrm{GeV}, \nonumber \\&\lambda '=2,\quad \lambda =\ -0.1, \end{aligned}$$

suggested by a no-scale model [35] in which the right-handed sneutrino is responsible for Starobinsky-like inflation. The value of $M_5$ is chosen so that we obtain the relic abundance corresponding to the cold dark matter density determined by Planck and other experiments [55]. As noted earlier, after setting $A_0 = B_0 = 0$, we no longer have the freedom of choosing $\tan \beta$ as a free parameter. For benchmark **B**, we find $\tan \beta \simeq 52$. The value of $M_5$ is large enough to satisfy LHC bounds from supersymmetric particle searches and the lightest Higgs mass is $m_h = 125.0 \pm 3.1$ GeV when calculated with the FeynHiggs code [56–59], which is comfortably consistent with the joint ATLAS and CMS measurement of $m_h$ [60]. It is important to compare correctly theoretical predictions of $B_s \rightarrow \mu ^+ \mu ^-$ with its experimental value, as was reviewed in [61]. In particular, one should compare the “untagged” computed value, instead of the tagged one, to its experimental counterpart. The relevance of this comparison for some supersymmetric scenarios was studied in [62], where it was pointed out that this difference is important for evaluating the validity of some scenarios. Using the SUSY_FLAVOR code [63–65] and the latest hadronic observables, we find that for the benchmark point under consideration the tagged value is $3.42 \times 10^{-9}$, whilst using a modified version of the SUSY_FLAVOR code we find that the untagged value is $3.76 \times 10^{-9}$. Because $\tan \beta$ is relatively high for this benchmark point, the branching ratio of $B_s \rightarrow \mu ^+ \mu ^-$ is somewhat large, but within the experimental 95 % CL upper limit [66, 67].

### Runnings of SU(5) parameters

**Fig. 1:**
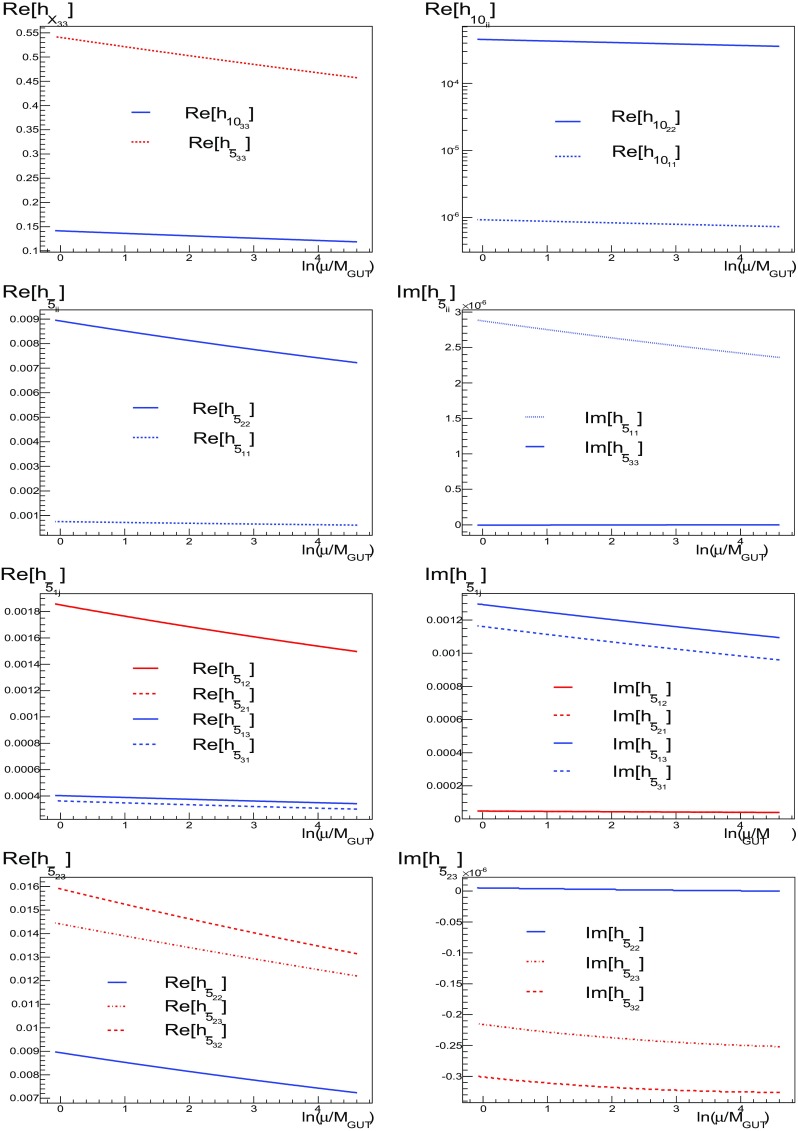
The runnings of the Yukawa couplings from $M_{ in }$ to $M_{\mathrm{GUT}}$ for the benchmark point $\mathbf {B}$, using the patterns specified via Ansatz A1. Note that the lines for $h_{\bar{5}_{12}}$ and $h_{\bar{5}_{21}}$ lie on *top* of each other

As already emphasised, the soft supersymmetry-breaking mass parameters are zero at the input scale in a no-scale model, but running between $M_{ in }$ and $M_{\mathrm{GUT}}$ leads in general to non-zero masses for both diagonal and non-diagonal elements. In particular, the latter are induced by the non-diagonal Yukawa couplings assumed in Ansatz A1. The runnings of the Yukawa couplings for benchmark **B** are shown in Fig. 1. Recall that we have assumed diagonal Yukawa matrices for the up-quark sector and the **10** of SU(5), and therefore we show only the evolution of the real part of $h_{10 ii}$. In contrast, for the ${\varvec{\bar{5}}}$ of SU(5), the Yukawa matrices are determined from (14) using Ansatz A1. Note that, with this definition, these Yukawa matrices are in general not symmetric (though the runnings of $h_{5_{12}}$ and $h_{5_{21}}$ are indistinguishable in the figure). The figure shows the runnings of the Yukawa couplings from the input scale $M_{ in }$ ($\ln (\mu /M_\mathrm{GUT}) \approx 4.6$ for benchmark **B**) down to the GUT scale ($\ln (\mu /M_\mathrm{GUT}) = 0$).

**Fig. 2:**
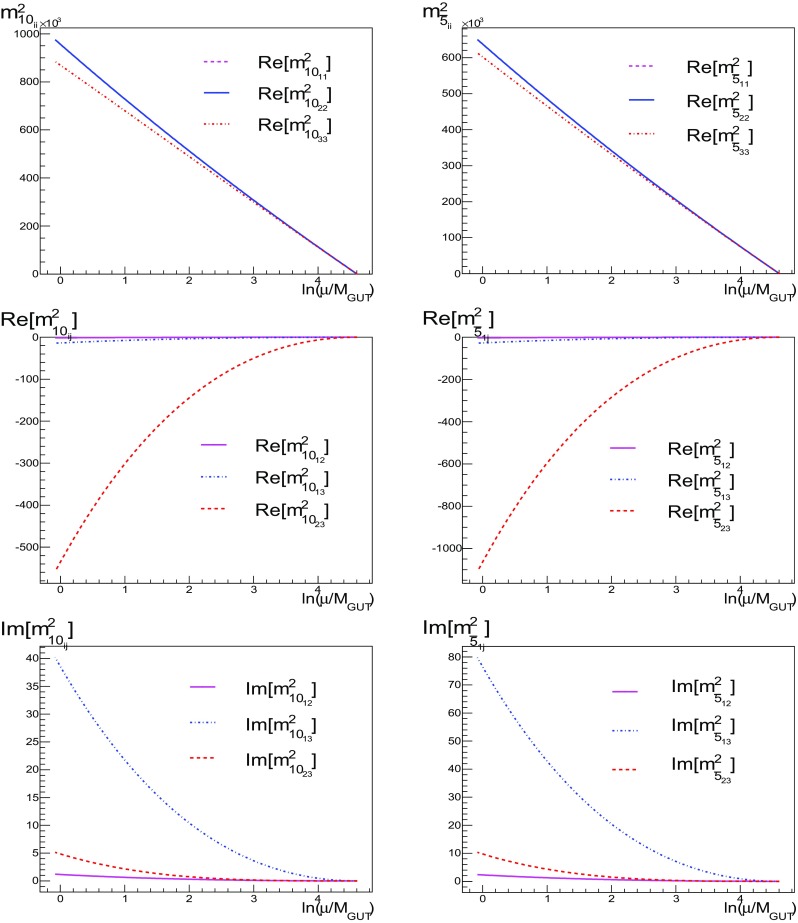
The runnings of diagonal (*left*) and off-diagonal (*right*) soft masses-squared associated with the **10** (*left*) and $\mathbf {\overline{5}}$ (*right*) representations in the SU(5) model between $M_{ in }$ and $M_{\mathrm{GUT}}$ for the benchmark point $\mathbf {B}$ (in units of GeV$^2$) using Ansatz A1 for the Yukawa couplings

The running of the soft scalar masses is shown in Fig. 2, where we have again assumed benchmark point **B** and ansatz A1. The top panels show the running of the diagonal soft masses for the **10** and ${\varvec{\bar{5}}}$ of SU(5). The middle panels show the real parts of the off-diagonal entries, and the bottom panels show the imaginary parts of the same off-diagonal entries (as these are hermitian quantities, information on the transposed entries are already given by the real and imaginary parts). All of the squark masses begin their evolution with $m^2 = 0$ at $M_{ in }$. The running of the diagonal components is driven by the value of the gaugino mass, here set at $M_{1/2}=1500$ GeV. We clearly see from Fig. 2 that the off-diagonal elements of the squark mass matrices induced by the non-diagonal Yukawa matrices remain very small after we have imposed the no-scale boundary conditions: we find $(m^2_{\bar{5}})_{12}, (m^2_{\widetilde{10}})_{12} \ll (m^2_{\bar{5}})_{13} \approx (m^2_{\widetilde{10}})_{13}\approx -20 \ \mathrm {GeV}^2$, $(m^2_{\bar{5}})_{23}\approx -1100 \ \mathrm {GeV}^2, \quad (m^2_{\widetilde{10}})_{23}\approx -550 \ \mathrm {GeV}^2$, while $(m^2_{\bar{5}})_{ii}$ and $(m^2_{\widetilde{10}})_{ii}$ are of order $10^5$ and $10^6$ GeV$^2$, respectively. Later we will use this evolution to place constraints on the size of the possible sizes of the off-diagonal elements at $M_{ in }$.

### Running of MSSM parameters

**Fig. 3:**
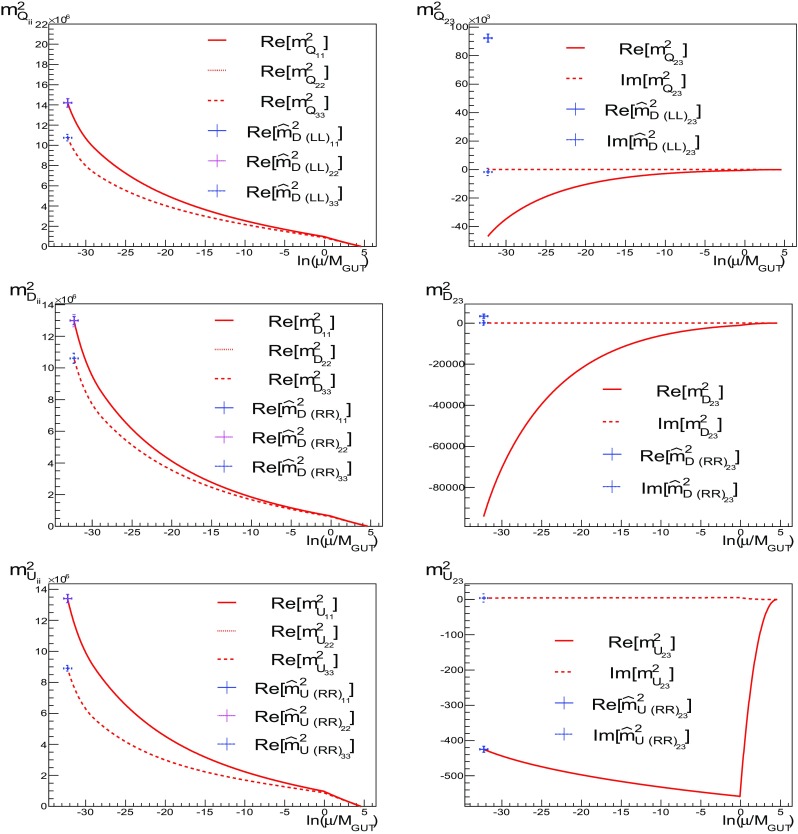
The runnings of soft-squared masses for the MSSM parameters for Ansatz A1. The *horizontal axis* is $\ln (\mu /M_{\mathrm{GUT}})$, and the *vertical axis* shows the mass-squared in units of [GeV]$^2$. For the diagonal soft-squared masses, the split between first and second generation is not appreciable on the scale of the plot. In each panel we show the runnings of ${m^2_{ Q}}_{ii}$, $i=1, 2, 3$, ${m^2_{ Q}}_{23}$, ${m^2_{D}}_{ii}$, $i=1, 2, 3$, ${m^2_{D}}_{23}$, ${m^2_{U}}_{ii}$, $i=1, 2, 3$ and ${m^2_{U}}_{23}$. The *red lines* in the plots run from the input scale, $M_{ in }$, down to $M_{ EW }$ (with the appropriate matching at $M_{ GUT }$) and are given in the basis where Yukawa couplings *are not* diagonal. The *blue crosses* show the result (at the electroweak scale) of running in the basis where the Yukawa matrices are diagonal. The states labelled without a hat are the states given in the basis where Yukawa couplings *are not* diagonal. The SU(5) parameters were specified in the basis where the Yukawa couplings are not diagonal

**Fig. 4:**
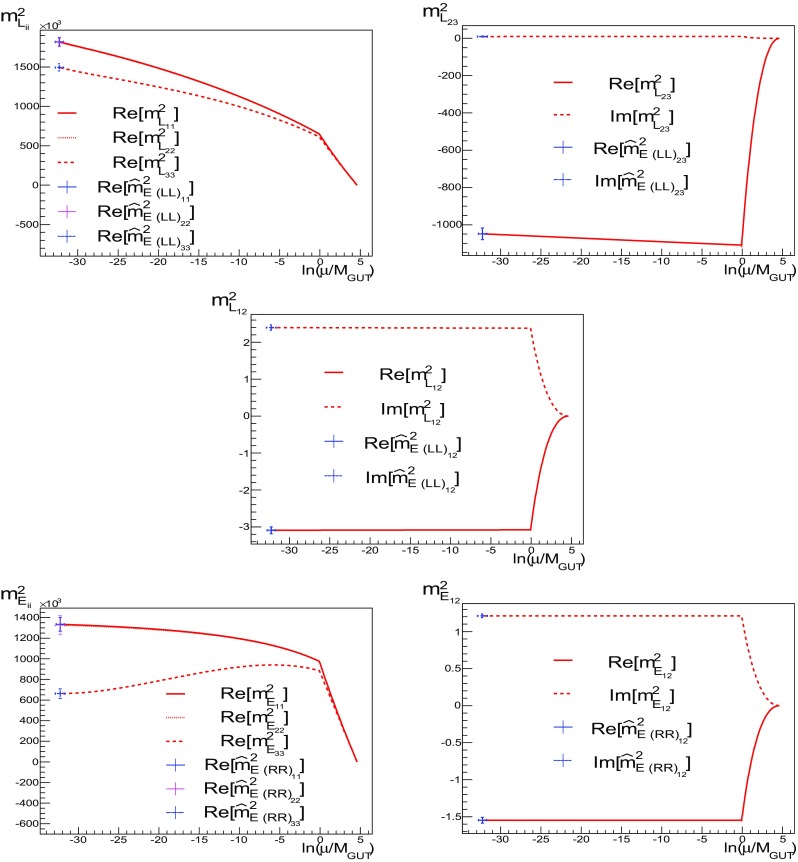
As in Fig. 3, but for ${m^2_{L}}_{ii}$, $i=1, 2, 3$, ${m^2_{L}}_{23}$, ${m^2_{L}}_{12}$, ${m^2_{E}}_{ii}$, $i=1, 2, 3$, and ${m^2_{E}}_{12}$. The *horizontal axis* is again $\ln (\mu /M_{\mathrm{GUT}})$

**Fig. 5:**
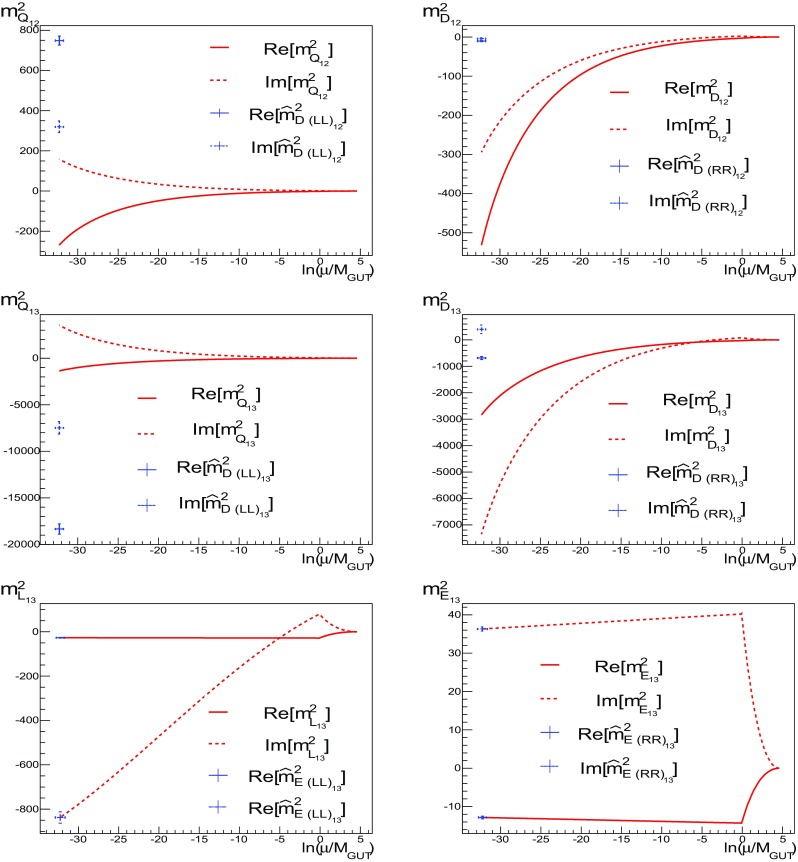
As in Fig. 4 but now for ${m^2_{ Q}}_{12}$, ${m^2_{D}}_{12}$ and ${m^2_{f}}_{13}$, $f=Q, D, L, E$. The *horizontal axis* is again $\ln (\mu /M_{\mathrm{GUT}})$

The matching of couplings and masses at $M_{\mathrm{GUT}}$, using (9), is made in the basis where the Yukawa couplings are not diagonal, i.e., we assume ansatz A1. The transformations to the super-CKM (SCKM) basis, where the Yukawa couplings are diagonal, are given by

20 $$\begin{aligned} \hat{m}^2_{ D (\mathrm{LL})}= & {} V^{D\dagger }_L m^2_{Q} V^D_L,\nonumber \\ \hat{m}^2_{ E (\mathrm{LL})}= & {} V^{E\dagger }_L m^2_{ L} V^E_L,\nonumber \\ \hat{m}^2_{ f (\mathrm RR)}= & {} V^{f\dagger }_R m^2_f V^f_R,\quad f=D,E,\nonumber \\\hat{m}^2_{ f (\mathrm LR)}= & {} -\hat{a}_f v_f + \mu \tan \beta \ \hat{m}_{f_i}\delta _{ij},\quad \nonumber \\ \hat{a}_f= & {} V^{f\dagger }_L a_f V^{f}_R, \quad f=D,E, \end{aligned}$$

where the matrices $V^{f\dagger }_X$ ($X=L,R$) are defined in (14) and the $\hat{m}_{f_i}$ are the fermion masses, $i=1,2,3$. We could also define $\hat{m}^2_{ U (\mathrm{LL})}=V^{U\dagger }_L m^2_{Q} V^U_L$, but we are taking $V^U_L$ to be diagonal. We note that SU(2)$_L$ invariance in the SCKM basis is preserved trivially, because then $m^2_{ Q}=V^D_L \hat{m}^2_{D (\mathrm{LL})} V^{D\dagger }_L$= $V^U_L \hat{m}^2_{U (\mathrm{LL})} V^{U\dagger }_L$ and so $\hat{m}^2_{ U (\mathrm{LL})}=V_\mathrm{{CKM}} \hat{m}^2_{D (\mathrm{LL})}V^{\dagger }_\mathrm{{CKM}}$. A more complete set of transformation rules are given in Appendix B. The choice of the Ansatz in Eqs. (15)–(18) induces off-diagonal entries at $M_{\mathrm{GUT}}$ in the SCKM basis, which are constrained by flavour-violating processes. In particular, for Ansätze 3 and 4, we find that $\text {BR}(\mu \rightarrow e\gamma )$ is too large and, in addition, for Ansatz 4 some electric dipole moments (EDMs) are too large. On the other hand, both the Ansätze A1 and A2 induce acceptable amounts of flavour violation. Ansatz 1 is interesting because both the right- and left-diagonalisation matrices $V_{L, R}$ are CKM-like. We plot the runnings of the MSSM soft-squared parameters for this Ansatz in Figs. 3, 4 and 5.

We plot in the first panel of Fig. 3 the running of some squark soft masses-squared. The horizontal axis is $x=\ln (\mu /M_{\mathrm{GUT}})$, and the red curves represent the evolution of states in the basis where Yukawa couplings *are not* diagonal. That is, starting with our boundary conditions: $m_0 = 0$ at $x \simeq 4.6$, the sfermion masses are run down to $M_{\mathrm{GUT}}$ where they are matched to the MSSM sfermion masses. As the running to $M_{\mathrm{GUT}}$ has induced some off-diagonal entries, these are also run down to the weak scale ($x \simeq -32$). This running is shown by the set of red curves. For example, at the electroweak scale, the running of the diagonal left-handed squark masses-squared ${m^2_{ Q}}_{33}$ reaches $11\times 10^6$ GeV$^2$, while ${m^2_{ Q}}_{ii\ne 3}$ reaches $14\times 10^6$ GeV$^2$. The split between the first and second generation is not appreciable on the scale displayed on the plot. We can diagonalise the mass matrices at the weak scale using (20) with the *V*’s defined by A1. That result is shown by the blue crosses. In the upper left panel it makes no difference whether we run in the diagonal (SCKM) basis (20) or in the non-diagonal Yukawa basis, as the blue crosses sit at the endpoint of the red curves.

In the second to sixth panels, we show the running of ${m^2_{ Q}}_{23}$, ${m^2_{D}}_{ii}$, $i=1,2,3$, ${m^2_{D}}_{23}$, ${m^2_{U}}_{ii}$, $i=1, 2, 3$, and ${m^2_{U}}_{23}$.

We again show as blue crosses the endpoints of the running at the weak scale when the states are in the basis where Yukawa couplings are diagonal. The positions of these show the impact of the changes in size of off-diagonal parameters. We see that the blue crosses differ only slightly from the running shown by the red curves for the imaginary parts of $Q_{23}$ and $D_{23}$. Since the Yukawa couplings are chosen to be diagonal for *U*, the blue crosses are found at the endpoints of the red curves in these cases.

In Figs. 4 and 5 we show the same for the runnings of ${m^2_{L}}_{ii}$, $i=1,2,3$, ${m^2_{L}}_{23}$, ${m^2_{L}}_{12}$, ${m^2_{E}}_{ii}$, $i=1, 2, 3$, ${m^2_{E}}_{12}$, ${m^2_{ Q}}_{12}$, ${m^2_{D}}_{12}$ and ${m^2_{f}}_{13}$, $f=Q, D, L, E$. We do not show the runnings of ${m^2_{E}}_{23}$, ${m^2_{U}}_{12}$ and ${m^2_{U}}_{13}$ because, given the matching conditions at $M_{\mathrm{GUT}}$, (9), and the fact that in the *E* and *U* sectors the Yukawa couplings are chosen to be diagonal, their runnings are similar to ${m^2_{U}}_{23}$, ${m^2_{E}}_{12}$ and ${m^2_{E}}_{13}$, respectively.

As one can see in Figs. 4 and 5, there is considerably less running for the (12) components of the squark mass matrices. For the (13) sectors, looking at Fig. 5, we find that, depending on the sector, the running can be more or less important. As expected for ${m^2_{E}}_{13}$, the running is negligible because in this sector the Yukawa couplings are chosen to be diagonal. Note that, due to the CP-violating phase of the CKM, the running of the imaginary parts of ${m^2_{D}}_{13}$ and ${m^2_{L}}_{13}$ become appreciable. In the *D* sector, however, once the SCKM transformations, (20), are taken into account, some of the CP violation is rotated away, as a result of the first condition of Ansatz 1, (15).

### Flavour-violating parameters

We define the flavour-violating parameters

21 $$\begin{aligned} (\delta _{ij}^f)_{\{ XY \} }\equiv \frac{ \hat{m}^2_{ f (XY)_{ij}} }{\sqrt{ \hat{m}^2_{ f (XX)_ {ii}} \hat{m}^2_{ f (YY)_{jj} }}},\quad f=D,E, \end{aligned}$$

where the mass matrices appearing on the right-hand side of the equation are defined in (20) and $X = L, R$. Flavour-violating parameters are often defined in the absence of a particular model in which the running can be performed explicitly. However, general limits on flavour-violating parameters cannot be obtained, because the forms in which they enter into observables are in general quite model-dependent; see for example [68, 69]. There are dependences both on the mass scales of the supersymmetric particles involved in a particular process – in a particular model not all the supersymmetric particles may be relevant – and on the specific underlying flavour framework. However, a few observables can severely constrain the parameters of (21) and give clean bounds on them, particularly for the sleptonic parameters. They still depend on the mass scale and assumptions of the underlying flavour model, but can be used as an indication, provided the model satisfies the conditions under which the bounds are derived. In particular, in [70] we find a set of conditions compatible with our assumptions, and we use them to compare to the lepton-flavour-violating parameters of (21).

**Fig. 6:**
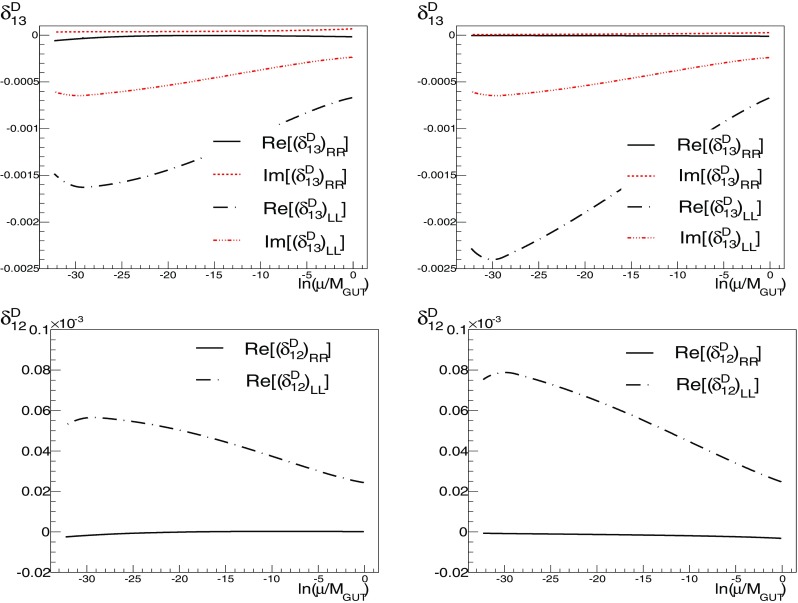
Comparison of the runnings of the *D*-quark flavour-violating parameters $\delta$ for A1 (*left panels*) and A2 (*right panels*), for $(\delta _{1i}^{D})_{\{ XX \}}$, $i=2,3$, $X=L,R$

The main purpose here in using the parameters of (21) is to compare our different models and to examine the different runnings and the contributions from the different sectors to a particular observable. Using the bounds of [70], we make comparisons and comment on cancellations in the models.

**Comparison between A1 and A2**

**Fig. 7:**
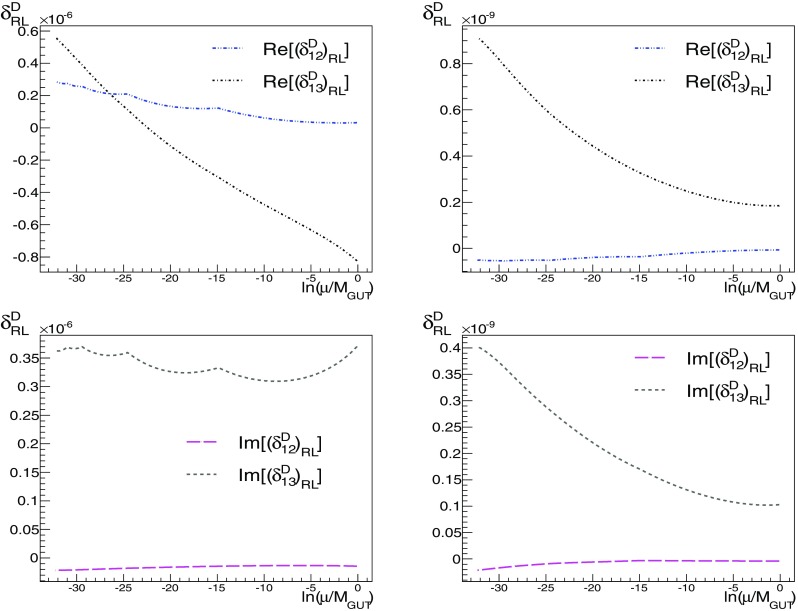
Comparison of the runnings of the *D*-quark flavour-violating parameters $\delta$ for A1 (*left panels*) and A2 (*right panels*), for $(\delta _{1i}^{D})_\mathrm{LR}$, $i=2,3$

The only difference between A1 and A2 is in the *D*-quark sector, Eqs. (15)–(18), so we concentrate our comparison on the *D*-squark sector (left and right). Our interest in comparing these Ansätze is to assess the relevance of switching off (A2) the effect of the CKM matrix in the right-handed *D* sector, and to check potential differences in the observables. The runnings of the flavour-violating parameters from the GUT scale to the weak scale is shown in Figs. 6 and 7 for models A1 and A2. At $M_{ GUT }$, the initial values for most of the $\delta$ parameters are quite similar for both these Ansätze. In some cases the sign differs, but they have a comparable absolute value.

The parameters that differ the most are the LR flavour-violating parameters in the (13) flavour sector, as can be seen from Tables 2 and 3. The behaviours of the real and imaginary parts are plotted in Fig. 7, notice the different scales of the plots. The differences between Ansätze A1 and A2 are to be expected, as they arise from the different choices for $V^D_R$. Looking at the terms that enter into the beta function of $a_D$, shown in (37) in Appendix B, we see that all terms involving $a_D$ are sensitive to the change of $V^D_R$, which affect directly the LR parameters. In contrast, we see from (36) of Appendix B that only some of the soft mass-squared transformations that contribute to the LL and RR parameters are sensitive to the choice of $V^D_R$. As a result, we do not expect the flavour-violating parameters coming entirely from the soft masses-squared to be very sensitive to the change of $V^D_R$. Indeed, looking at Tables 2 and 3, we see that the difference is at most one order of magnitude in the (13) RR and LL sectors for A1 and A2. In the (12) sector, we again see the strong dependence on $V^D_R$ in the LR parameters and relatively small changes in the RR and LL parameters. In the (23) sector, none of the terms are greatly affected by the choice of $V^D_R$. Because of the difference between the Yukawa couplings $h_{D_{23}}$ and $h_{D_{32}}$, the parameters $a_{D_{23}}$ and $a_{D_{32}}$ will have different values at the EW scale, but their absolute values are similar because their runnings are dominated by the largest Yukawa coupling, i.e., terms $\propto \hat{h}^2_{D_{3}}$ in (37), and contain $V^{D\dagger }_{L,R ii}$ elements. This is not the case for the lighter sectors, because their Yukawa couplings are smaller. By way of comparison, we plot the runnings of some of the (12) flavour parameters in Fig. 6.

Due to the running of the $a_D$ beta function due in particular to the third and fourth terms in (37), $a_D$ will not evolve symmetrically even if $h_D$ is symmetric, and therefore

22 $$\begin{aligned} \mathrm{{Re}}[(\delta ^D_{ij})_\mathrm{RL}]\ne & {} \mathrm{{Re}}[(\delta ^D_{ji})_\mathrm{RL}] \, , \nonumber \\ \mathrm{{Im}}[(\delta ^D_{ij})_\mathrm{RL}]\ne & {} \mathrm{{Im}}[(\delta ^D_{ji})_\mathrm{RL}] \, . \end{aligned}$$

If, in addition, $h_D$ is not symmetric (as in Ansatz A2), this effect can be quite noticeable (in particular for $(\delta ^D_{12})_\mathrm{RL}$, $(\delta ^D_{21})_\mathrm{RL}$, $(\delta ^D_{13})_\mathrm{RL}$ and $(\delta ^D_{31})_\mathrm{RL}$). However, the behaviours of the other flavour-violating parameters tells us that their evolutions in the SCKM basis do not differ too much, which is explained by the forms of the transformations in (36), so we have not plotted them in Figs. 6 and 7. The order-of-magnitude differences in the real and imaginary parts of $(\delta _{ij}^f)_{\{ XY \}}$ are given in Tables 2 and 3, respectively.

**Table 2:** Comparison of the real parts of the *D*-squark parameters $(\delta _{ij}^f)_{\{ XY \}}$ in Ansätze A1 and A2

	A1	A2	Bound (Process) [71]
$\mathrm{{Re}}[(\delta _{12}^D)_{ \mathrm RR }]$	$-$ $8 \times 10^{-7}$	$-$ $2\times 10^{-7}$	$10^{-2}$ ($\Delta M_K$)
$\mathrm{{Re}}[(\delta _{12}^D)_{ \mathrm{LL} }]$	$5 \times 10^{-5}$	$8\times 10^{-5}$	$10^{-2}$ ($\Delta M_K$)
$\mathrm{{Re}}[(\delta _{12}^D)_{ \{ \mathrm{LR}, \mathrm{RL} \} }]$	$3 \times 10^{-7}$	$-$ $5 \times 10^{-11}$	–
$\mathrm{{Re}}[(\delta _{21}^D)_{ \{ \mathrm{LR}, \mathrm{RL} \} }]$	$3 \times 10^{-7}$	$3 \times 10^{-7}$	–
$\mathrm{{Re}}[(\delta _{13}^D)_{ \mathrm RR }]$	$-$ $6\times 10^{-5}$	$-$ $2\times 10^{-6}$	–
$\mathrm{{Re}}[(\delta _{13}^D)_{ \mathrm{LL} }]$	$-$ $1\times 10^{-3}$	$-$ $2\times 10^{-3}$	–
$\mathrm{{Re}}[(\delta _{13}^D)_{ \{ \mathrm{LR}, \mathrm{RL } \} }]$	$5 \times 10^{-7}$	$9\times 10^{-10}$	–
$\mathrm{{Re}}[(\delta _{31}^D)_{ \{ \mathrm{LR}, \mathrm{RL } \} }]$	$-$ $5 \times 10^{-7}$	$-$ $5 \times 10^{-7}$	–
$\mathrm{{Re}}[(\delta _{23}^D)_{ \mathrm RR }]$	$3 \times 10^{-4}$	$-$ $8 \times 10^{-3}$	$10^{-2}$ ($\text {BR}(B_s\rightarrow \mu ^+\mu ^-)$)
$\mathrm{{Re}}[(\delta _{23}^D)_{ \mathrm{LL} }]$	$7\times 10^{-3}$	$1\times 10^{-2}$	$10^{-2}$ ($\text {BR}(B_s\rightarrow \mu ^+\mu ^-)$)
$\mathrm{{Re}}[(\delta _{23}^D)_{ \{ \mathrm{LR}, \mathrm{RL } \} }]$	$-$ $2\times 10^{-6}$	$-$ $3 \times 10^{-6}$	–
$\mathrm{{Re}}[(\delta _{32}^D)_{ \{ \mathrm{LR}, \mathrm{RL } \} }]$	$2\times 10^{-6}$	$2 \times 10^{-6}$	–

**Comparison between A1 and A4**

The difference between A1 and A4 is due to the replacement of $$ V^{E*}_R=V^E_L=1$$ by $$V^{E*}_R=V^E_L=V_\mathrm{{CKM}}$$, so we expect a significant increase in the leptonic *L*, *R* flavour-violating parameters, as they are directly linked to the trilinear couplings, which are enhanced by the running of the Yukawa couplings.

From Table 4 we can see that this is the case for all the real parts of the flavour-violating parameters, specially for $(\delta _{1j}^E)_{\{\mathrm{LR},\mathrm{RL}\}}$ for $j=2,3$. This can again be understood in terms of the evolution of the different terms entering into the beta function of the *E* trilinear terms. These have the same form of (37) with the replacement $D\rightarrow E$, and no analogous *U* terms since we are not considering neutrinos. We can see that in general all flavour-violating parameters coming entirely from the soft-squared masses (i.e., RR and LL) have a milder change than from the $\mathrm LR$ counterparts, as expected from the analogous form of the terms entering into the $m^2_E$ beta function (analogous to (36) with the proper replacements).

For A4, the parameter $|\mathrm {Re}[(\delta _{12}^E)_{ \mathrm{LL} }]|$ exceeds the limit of the general analysis of [70], and most of the other parameters are at their limits. The different orders of magnitude of the real and imaginary parts of $(\delta _{ij}^{L,E})_{\{ XY \}}$ are given in Tables 4 and 5, respectively.

**Fig. 8:**
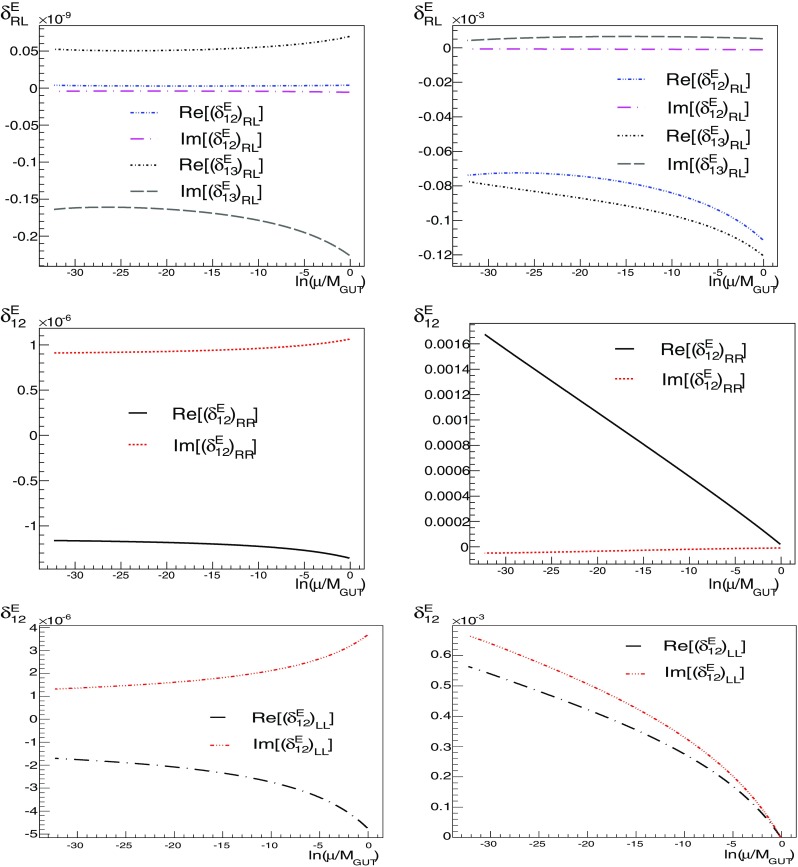
Comparison of the runnings of the *E*-lepton flavour-violating parameters $\delta$ for A1 (*left panels*) and A4 (*right panels*), for $(\delta _{1i}^{E})_\mathrm{LR}$, $i=2,3$, and $(\delta _{12}^{E})_{XX}$, $X=L,R$

In Fig. 8 we compare the runnings of the *E*-lepton flavour-violating parameters $\delta$ for A1 (left panels) and A4 (right panels), for $(\delta _{1i}^{E})_\mathrm{LR}$, $i=2,3$, and $(\delta _{12}^{E})_{XX}$, $X=L,R$. Although the transformation to the SCKM basis, where flavour-violating parameters are computed, has the effect of cancelling partially the effect of the running of soft-squared masses, it is not enough to suppress sufficiently $\text {BR}(\mu \rightarrow e\gamma )$, and in fact there is a significant increase in $\text {BR}(\tau \rightarrow e\gamma )$ and $\text {BR}(\tau \rightarrow \mu \gamma )$ with respect to Ansatz A1; see Tables 6–7.

In addition, from the imaginary parts of $|\mathrm{{Im}}[(\delta _{ij}^{D,E})_{ XY }]|$ we can get a significant contribution to the EDMs [72]. We have used the SUSY_FLAVOR code [63–65] to compute the EDMs, but we can understand easily how the imaginary parts of the flavour-violating parameters are constrained. The constrained combinations are

23 $$\begin{aligned} \delta ^f_{131}\equiv \mathrm{Arg}\left[ (\delta ^f_{13})_\mathrm{LL} (\delta ^f_{33})_\mathrm{LR} (\delta ^f_{31})_\mathrm{RR} \right] , \quad f=D,E. \end{aligned}$$

**Fig. 9:**
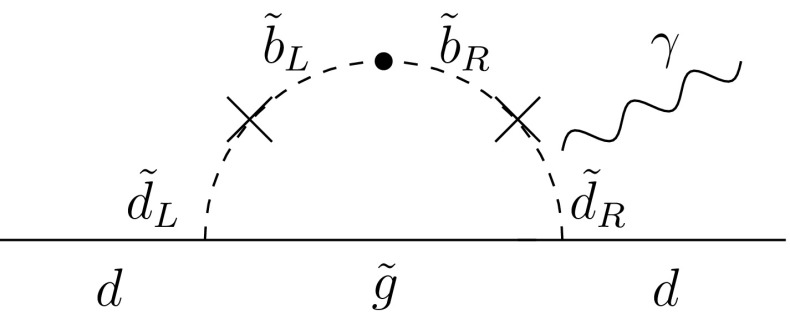
Contribution of flavour-violating process to the d-quark EDM

Since A1 and A2 differ in their *D* sectors, a direct difference in the values of the neutron EDM is expected (as reflected in Table 6). Note that, in Fig. 9, both the real and imaginary parts of $(\delta ^f_{13})_\mathrm{LL}$ and $(\delta ^f_{13})_\mathrm{RR}$ become important. Specifically, the leading term of the imaginary part of $(\delta ^f_{13})_\mathrm{LL} (\delta ^f_{33})_\mathrm{LR} (\delta ^f_{31})_\mathrm{RR}$ is

$$\begin{aligned}&\mathrm{{Im}}[(\delta ^f_{13})_\mathrm{LL}] \mathrm{{Re}}[(\delta ^f_{33})_\mathrm{LR}] \mathrm{{Re}}[(\delta ^f_{31})_\mathrm{RR}] \nonumber \\&+ \mathrm{{Re}}[(\delta ^f_{13})_\mathrm{LL}] \mathrm{{Re}}[(\delta ^f_{33})_\mathrm{LR}] \mathrm{{Im}}[(\delta ^f_{31})_\mathrm{RR}]. \nonumber \end{aligned}$$

**Table 3:** Comparison of the imaginary parts of the *D*-squark parameters $(\delta _{ij}^f)_{\{ XY \}}$ in Ansätze A1 and A2. The bounds on the imaginary parts in these scenarios cannot be taken directly from the literature, but must instead be constructed from different observables (see Sect. 3.4)

	A1	A2
$\mathrm{{Im}}[(\delta _{12}^D)_{ \mathrm RR }]$	$-3\times 10^{-7}$	$2\times 10^{-7}$
$\mathrm{{Im}}[(\delta _{12}^D)_{ \mathrm{LL} }]$	$2\times 10^{-5}$	$3\times 10^{-5}$
$\mathrm{{Im}}[(\delta _{12}^D)_{ \{ \mathrm{LR}, \mathrm{RL } \} }]$	$-2\times 10^{-8}$	$-2\times 10^{-11}$
$\mathrm{{Im}}[(\delta _{21}^D)_{ \{ \mathrm{LR}, \mathrm{RL } \} }]$	$5\times 10^{-9}$	$1 \times 10^{-8}$
$\mathrm{{Im}}[(\delta _{13}^D)_{ \mathrm RR }]$	$3 \times 10^{-5}$	$6\times 10^{-6}$
$\mathrm{{Im}}[(\delta _{13}^D)_{ \mathrm{LL} }]$	$-6\times 10^{-4}$	$-6\times 10^{-4}$
$\mathrm{{Im}}[(\delta _{13}^D)_{ \{ \mathrm{LR}, \mathrm{RL } \} }]$	$4\times 10^{-7}$	$4\times 10^{-10}$
$\mathrm{{Im}}[(\delta _{31}^D)_{ \{ \mathrm{LR}, \mathrm{RL } \} }]$	$-1\times 10^{-8}$	$-1\times 10^{-8}$
$\mathrm{{Im}}[(\delta _{23}^D)_{ \mathrm RR }]$	$1\times 10^{-5}$	$2\times 10^{-6}$
$\mathrm{{Im}}[(\delta _{23}^D)_{ \mathrm{LL} }]$	$-1 \times 10^{-4}$	$-1\times 10^{-4}$
$\mathrm{{Im}}[(\delta _{23}^D)_{ \{ \mathrm{LR}, \mathrm{RL } \} }]$	$8 \times 10^{-8}$	$3\times 10^{-9}$
$\mathrm{{Im}}[(\delta _{32}^D)_{ \{ \mathrm{LR}, \mathrm{RL } \} }]$	$-5 \times 10^{-8}$	$-5\times 10^{-8}$

**Table 4:** Comparison of the real parts of the leptonic parameters $(\delta _{ij}^E)_{\{ XY \}}$ in Ansätze A1 and A4

	A1	A4	Bound (Process) [70]
$\mathrm{{Re}}[(\delta _{12}^E)_{ \mathrm RR }]$	$-1 \times 10^{-6}$	$2 \times 10^{-3}$	$10^{-3}$ ($\mu \rightarrow e \gamma$)
$\mathrm{{Re}}[(\delta _{12}^E)_{ \mathrm LL }]$	$-2 \times 10^{-6}$	$6\times 10^{-4}$	$10^{-5}$ ($\mu \rightarrow e \gamma$)
$\mathrm{{Re}}[(\delta _{12}^E)_{ \{ \mathrm{LR}, \mathrm{RL } \} }]$	$4\times 10^{-12}$	$-7\times 10^{-5}$	$10^{-5}$ ($\mu \rightarrow e \gamma$)
$\mathrm{{Re}}[(\delta _{21}^E)_{ \{ \mathrm{LR}, \mathrm{RL } \} }]$	$2\times 10^{-9}$	$-7\times 10^{-5}$	$10^{-5}$ ($\mu \rightarrow e \gamma$)
$\mathrm{{Re}}[(\delta _{13}^E)_{ \mathrm RR }]$	$-1\times 10^{-5}$	$-5\times 10^{-3}$	$10^{-2}$ ($\tau \rightarrow e \gamma$)
$\mathrm{{Re}}[(\delta _{13}^E)_\mathrm{LL }]$	$-2\times 10^{-5}$	$-2\times 10^{-3}$	$10^{-3}$ ($\tau \rightarrow e \gamma$)
$\mathrm{{Re}}[(\delta _{13}^E)_{ \{ \mathrm{LR}, \mathrm{RL } \} }]$	$5\times 10^{-11}$	$-8 \times 10^{-5}$	$10^{-2}$ ($\tau \rightarrow e \gamma$)
$\mathrm{{Re}}[(\delta _{31}^E)_{ \{ \mathrm{LR}, \mathrm{RL } \} }]$	$3\times 10^{-9}$	$-9 \times 10^{-5}$	$10^{-2}$ ($\tau \rightarrow e \gamma$)
$\mathrm{{Re}}[(\delta _{23}^E)_{ \mathrm RR }]$	$-5\times 10^{-4}$	$2\times 10^{-2}$	$10^{-2}$ ($\tau \rightarrow \mu \gamma$)
$\mathrm{{Re}}[(\delta _{23}^E)_\mathrm{LL }]$	$-6\times 10^{-4}$	$7\times 10^{-3}$	$10^{-2}$ ($\tau \rightarrow \mu \gamma$)
$\mathrm{{Re}}[(\delta _{23}^E)_{ \{ \mathrm{LR}, \mathrm{RL } \} }]$	0	$5\times 10^{-4}$	$10^{-2}$ ($\tau \rightarrow \mu \gamma$)
$\mathrm{{Re}}[(\delta _{32}^E)_{ \{ \mathrm{LR}, \mathrm{RL } \} }]$	0	$5\times 10^{-4}$	$10^{-2}$ ($\tau \rightarrow \mu \gamma$)

**Table 5:** Comparison of the imaginary parts of the leptonic parameters $(\delta _{ij}^f)_{\{ XY \}}$ in Ansätze A1 and A4

	A1	A4
$\mathrm{{Im}}[(\delta _{12}^E)_{ \mathrm RR }]$	$9\times 10^{-7}$	$-5\times 10^{-5}$
$\mathrm{{Im}}[(\delta _{12}^E)_\mathrm{LL }]$	$1\times 10^{-6}$	$7\times 10^{-4}$
$\mathrm{{Im}}[(\delta _{12}^E)_{ \{ \mathrm{LR}, \mathrm{RL } \} }]$	$-4\times 10^{-12}$	$-6\times 10^{-7}$
$\mathrm{{Im}}[(\delta _{21}^E)_{ \{ \mathrm{LR}, \mathrm{RL } \} }]$	$2\times 10^{-9}$	$-6\times 10^{-6}$
$\mathrm{{Im}}[(\delta _{13}^E)_{ \mathrm RR }]$	$4\times 10^{-5}$	$4\times 10^{-4}$
$\mathrm{{Im}}[(\delta _{13}^E)_\mathrm{LL }]$	$-5\times 10^{-4}$	$-3\times 10^{-3}$
$\mathrm{{Im}}[(\delta _{13}^E)_{ \{ \mathrm{LR}, \mathrm{RL } \} }]$	$-2\times 10^{-10}$	$4\times 10^{-6}$
$\mathrm{{Im}}[(\delta _{31}^E)_{ \{ \mathrm{LR}, \mathrm{RL } \} }]$	$3\times 10^{-5}$	$2\times 10^{-4}$
$\mathrm{{Im}}[(\delta _{23}^E)_{ \mathrm RR }]$	$5\times 10^{-6}$	$6\times 10^{-5}$
$\mathrm{{Im}}[(\delta _{23}^E)_\mathrm{LL }]$	$6\times 10^{-6}$	$-8\times 10^{-4}$
$\mathrm{{Im}}[(\delta _{23}^E)_{ \{ \mathrm{LR}, \mathrm{RL } \} }]$	0	$9\times 10^{-7}$
$\mathrm{{Im}}[(\delta _{23}^E)_{ \{ \mathrm{LR}, \mathrm{RL } \} }]$	0	$4\times 10^{-5}$

In [72] sensitivities for the quantities $\delta ^f_{131}$ defined in (23) were computed using the mass-insertion approximation with a common scale for soft masses of 1 TeV. It was found, in particular, that $\delta ^f_{131}\sim 10^{-4}-10^{-3}$. Since our model has specific and correlated values for the soft parameters, we can compare the impacts of $(\delta ^f_{13})_\mathrm{LL}$ and $(\delta ^f_{13})_\mathrm{RR}$ directly to the neutron EDM. We note that the imaginary parts of $(\delta ^f_{13})_\mathrm{LL}$ and $(\delta ^f_{13})_\mathrm{RR}$ in A2 found in Table 3 are approximately one order of magnitude smaller than the corresponding parameters in A1. Hence the neutron EDM is slightly decreased (by less than an order of magnitude), as seen in Table 6. Using these results, in Sect. 4 we place bounds on $(\delta ^f_{13})_\mathrm{LL}$ and $(\delta ^f_{13})_\mathrm{RR}$ by saturating the EDM bound.

### Comments on the results and comparison to observables 

Ansätze A1 and A2 predict acceptable flavour violation, while Ansätze A3 and A4 do not. We recall that the properties of the *D* and *L* sectors are controlled by the $\bar{\mathbf {5}}$ sector of SU(5), whereas the *Q*, *U* and *E* sectors are controlled by the $\mathbf {10}$ sector. The premise of the Ansatz A4 for the Yukawa couplings, (18), was that if soft mass-squared sectors were transformed to the SCKM basis by the same transformations as the corresponding Yukawa sectors, then the off-diagonal parameters of the corresponding sectors would be suppressed because the off-diagonal elements of $m^2_{f}$ would be mainly rotated away with the same matrices that make the Yukawa couplings diagonal. This is largely the case for the *D* sector: in the cases of $(m^2_{f})_{13}$ and $(m^2_{f})_{23}$, for $f=D$, the rotation to the SCKM matrix produces a smaller matrix element than in the non-SCKM basis. However, this is not the case for other sectors, where too much flavour violation is produced. On the other hand, in the cases of Ansätze A2 and A1, the rotation to the SCKM basis effectively rotates away any large flavour violation.

For A1, with the $E_\mathrm{LL}$ sector we expected a similar behaviour (because the sector is directly linked to the diagonalising matrices of $h_E$), but the rotation away of parameters is not as successful as in the *D* sector (see Fig. 8). For the sectors *Q*, *U* and *E*, associated with the 10 sector of SU(5), which is treated as having diagonal matrices, the off-diagonal elements of $m^2_{f}$ for $f=\ Q,\ D,\ U$ appear as a consequence of the off-diagonal elements of $m^2_{f}$ for $f=E, L$. So they are not related, in principle, but since the Yukawa couplings $h_d$ and $h_e$ are related, we expected that the SCKM transformations of the soft sectors *Q* and *E* would tend also to suppress the off-diagonal elements. This is, however, not the case for them, specially for the *E* sector, where the off-diagonal elements may be considerably enlarged, as seen in Fig. 8.

**Table 6:** Comparison of the predictions for Ansätze A1 and A2 with the experimental values. The values have been obtained with the SUSY_FLAVOR code. Without flavour violation, we obtain $\text {BR}(B\rightarrow X_s\gamma )=4.01\times 10^{-4}$. The value in the table, which includes flavour violation, is consistent with experiment within $2\sigma$, taking into account the Standard Model uncertainty of $0.23\times 10^{-4}$. The NNLO Standard Model value of $|\epsilon _K|$ is $(1.81\pm 0.28)\times 10^{-3}$. See Sect. 3.4 for notes regarding the values of $\Delta m_K$ and $\Delta m_{B_s}$

Relevant observables for A1 and A2			
	Experimental values		
EDMs (e cm)		A1	A2
Electron EDM	$8.7 \times 10^{-29}$ [47]	$1.11\times 10^{-30}$	$1.19\times 10^{-30}$
Muon EDM	$-(0.1 \pm 0.9) \times 10^{-19}$ [47]	$3.49\times 10^{-30}$	$3.63\times 10^{-30}$
Tau EDM	$-1.27\times 10^{-25}$	$6.92\times 10^{-30}$	$7.13\times 10^{-30}$
Neutron EDM	$<2.9 \times 10^{-26}$ [48] $O(10^{-28})$ [73]	$-1.17\times 10^{-28}$	$-7.18\times 10^{-29}$
M. Anomalies			
$\Delta a_e$	$8.2\times 10^{-13}$ [74]	$1.43\times 10^{-14}$	$1.43 \times 10^{-14}$
$\Delta a_\mu$	$(2.87 \pm 0.80)\times 10^{-8}$	$6.15\times 10^{-10}$	$6.16 \times 10^{-10}$
$\Delta a_\tau$	$(-5.3 \times 10^{-2}, 1.2 \times 10^{-3})$	$1.80\times 10^{-7}$	$1.80\times 10^{-7}$
$l^j \rightarrow l^i \gamma$ decays			
$\text {BR}(\mu \rightarrow e\gamma )$	$5.7 \times 10^{-13}$ [75]	$5.3 \times 10^{-16}$	$5.4 \times 10^{-16}$
$\text {BR}(\tau \rightarrow e\gamma )$	$3.3\times 10^{-8}$ [76]	$1.2 \times 10^{-13}$	$1.2 \times 10^{-13}$
$\text {BR}(\tau \rightarrow \mu \gamma )$	$4.4\times 10^{-8}$ [76]	$6.0 \times 10^{-12}$	$6.2 \times 10^{-12}$
B decays			
$\text {BR}(B_s\rightarrow \mu ^+\mu ^-)$	$(2.9\pm 0.7 \times 10^{-9})$ [66]	$3.4\times 10^{-9}$	$3.4\times 10^{-9}$
Untagged		$3.75\times 10^{-9}$	$3.76\times 10^{-9}$
$\text {BR}(B_d\rightarrow \mu ^+\mu ^-)$	$(3.6^{+1.6}_{-1.4}) 10^{-10}$ [66]	$1.1\times 10^{-10}$	$1.0\times 10^{-10}$
$\text {BR}(B_s\rightarrow \mu ^+e^-)$	$2.0 \times 10^{-7}$	$2.09\times 10^{-27}$	$2.13\times 10^{-27}$
$\text {BR}(B \rightarrow \tau \nu )$	$(1.20 \pm 0.25)\times 10^{-4}$	$7.43 \times 10^{-5}$	$7.43 \times 10^{-5}$
$\text {BR}(B\rightarrow X_s\gamma )$	$(3.55 \pm 0.24 \pm 0.09)\times {10}^{{-4}}$ [77]	$3.92 \times 10^{-4}$	$3.92 \times 10^{-4}$
$\nu$ Kaon decays			
$\text {BR}(K_L^0 \rightarrow \pi ^0 \nu \nu )$	$2.6\times 10^{-8}$	$2.32\times 10^{-11}$	$2.32\times 10^{-11}$
$\text {BR}(K^+ \rightarrow \pi ^+ \nu \nu )$	$(1.7\pm 1.1) \times 10^{-10}$	$7.64\times 10^{-11}$	$7.64\times 10^{-11}$
$$\mathrm {KK\ mixing}$$			
$|\epsilon _K|$	$(2.223\pm 0.010)10^{-3}$	$1.81\times 10^{-3}$	$1.81\times 10^{-3}$
$\Delta m_K$ (GeV)	$(3.63\pm 0.0059) \times 10^{-15}$	$2.63 \times 10^{-15}$	$2.63 \times 10^{-15}$
BB mixings			
$\Delta m_{B_d}$	$(3.36\pm 1.97 \times 10^{-2})\times 10^{-13}$	$3.05\times 10^{-13}$	
$\Delta m_{B_s}$	$(1.164\pm 1.4\times 10^{-3})\times 10^{-11}$	$0.98\times 10^{-11}$	

**Table 7:** Comparison of the predictions for our Ansätze A3 and A4 with the experimental values

Relevant observables for A3 and A4			
	Experimental values		
EDMs (e cm)		A3	A4
Electron EDM	$8.7 \times 10^{-29}$ [47]	$6.42\times 10^{-28}$	$2.7\times 10^{-27}$
Muon EDM	$-(0.1 \pm 0.9) \times 10^{-19}$ [47]	$-1.40\times 10^{-28}$	$-3.0\times 10^{-27}$
Tau EDM	$-1.27\times 10^{-25}$	$4.57\times 10^{-28}$	$3.7\times 10^{-28}$
Neutron EDM	$$ <2.9 \times 10^{-26}$$ [48] $$O(10^{-28}) $$ [73]	$$-3.59\times 10^{-29}$$	$$3.7\times 10^{-30}$$
M. Anomalies			
$\Delta a_e$	$8.2\times 10^{-13}$ [74]	$3.2\times 10^{-14}$	$1.2 \times 10^{-14}$
$\Delta a_\mu$	$(2.87 \pm 0.80)\times 10^{-8}$	$1.4\times 10^{-9}$	$6.1 \times 10^{-10}$
$\Delta a_\tau$	$(-5.3 \times 10^{-2}, 1.2 \times 10^{-3})$	$4.1\times 10^{-7}$	$1.8\times 10^{-7}$
$l^j \rightarrow l^i \gamma$ decays			
$\text {BR}(\mu \rightarrow e\gamma )$	$5.7 \times 10^{-13}$ [75]	$1.7 \times 10^{-9}$	${7.49 \times 10^{-10}}$
$\text {BR}(\tau \rightarrow e\gamma )$	$3.3\times 10^{-8}$ [76]	$6.6 \times 10^{-13}$	$1.02 \times 10^{-12}$
$\text {BR}(\tau \rightarrow \mu \gamma )$	$4.4\times 10^{-8}$ [76]	$1.05 \times 10^{-11}$	$1.52 \times 10^{-12}$
B decays			
$\text {BR}(B_s\rightarrow \mu ^+\mu ^-)$	$(2.9\pm 0.7 \times 10^{-9})$ [66]	$3.3\times 10^{-9}$	$3.4\times 10^{-9}$
Untagged		$3.66\times 10^{-9}$	$3.75\times 10^{-9}$
$\text {BR}(B_d\rightarrow \mu ^+\mu ^-)$	$(3.6^{+1.6}_{-1.4}) 10^{-10}$ [66]	$1.4\times 10^{-10}$	$1.1\times 10^{-10}$
$\text {BR}(B_s\rightarrow \mu ^+e^-)$	$2.0 \times 10^{-7}$	$9.1\times 10^{-23}$	$2.3\times 10^{-21}$
$\text {BR}(B \rightarrow \tau \nu )$	$(1.20 \pm 0.25)\times 10^{-4}$	$7.37 \times 10^{-5}$	$7.43 \times 10^{-5}$
$\text {BR}(B\rightarrow X_s\gamma )$	$(3.55 \pm 0.24 \pm 0.09)\times 10^{-4}$ [77]	$4.22 \times 10^{-4}$	$3.9 \times 10^{-4}$
$\nu$ Kaon decays			
$\text {BR}(K_L^0 \rightarrow \pi ^0 \nu \nu )$	$2.6\times 10^{-8}$	$2.32\times 10^{-11}$	$2.3\times 10^{-11}$
$\text {BR}(K^+ \rightarrow \pi ^+ \nu \nu )$	$(1.7\pm 1.1) \times 10^{-10}$	$7.6\times 10^{-11}$	$7.6\times 10^{-11}$
$$ \mathrm {KK\ mixing}$$			
$|\epsilon _K|$	$(2.223\pm 0.010)10^{-3}$	$1.82\times 10^{-3}$	$1.81\times 10^{-3}$
$\Delta m_K$ (GeV)	$(3.483\pm 0.0059)\times 10^{-15}$	$2.63\times 10^{-15}$	$2.63\times 10^{-15}$

In Tables 6 and 7 we compare the values of the relevant observables predicted by the Ansätze (15)–(18), and the corresponding current experimental values.

The CP-violating parameter $\epsilon '$ could give important constraints on models where LR flavour-violating contributions are much bigger than their RR and LL counterparts: $|(\delta ^D_{ij})_\mathrm{RL}|\gg$ $|(\delta ^D_{ij})_{XX}|$, $X=R, L$. This is not the case in our framework, where both the real and imaginary parts of $[(\delta ^D_{ij})_{XX}]$ are much bigger than $[(\delta ^D_{ij})_\mathrm{RL}]$. Furthermore, chirality-conserving mass-insertion parameters turn out to be more stringently constrained from $\Delta m_K$ and $\epsilon _K$ [78], which enter as combinations of the real and imaginary parts of $(\delta ^D_{ij})_{XX}$ for the combinations $ij=\{12,21\}$ [79]. However, regarding $\Delta m_K$, even in the Standard Model precise computations are not possible due to unknown long-distance contributions. Hence, we compare the best estimate obtained from the short-distance (SD) contributions [80], denoted by $\Delta m^\mathrm{SD}_K=(3.1\pm 1.2 )\times 10^{-15}$, to the experimental value, see Table 7. We see that the central value of $\Delta m^\mathrm{SD}_K$ accounts for 86 % of the experimental central value, and its uncertainty can easily account for the reported experimental value within $1\sigma$. The value that we obtain in our model also lies comfortably within $1\sigma$ of the experimental value, taking into account the SD uncertainty. For $\Delta m_{B_s}$, the SM value is $(17.70\pm 15\,\%)$, and the value that we obtain is in better agreement with the experimental value.

## Beyond no-scale GUTs: maximal flavour violation 

In this section we explore the possibilities for deformations of the pure no-scale boundary conditions with non-vanishing scalar masses. We study the conditions under which flavour violation could be maximal, in the sense that off-diagonal entries in the scalar mass matrices could be as large as the diagonal entries at some super-GUT input scale $M_{ in }> M_{ GUT }$. We emphasise that, as in the previous section, we are not trying to explain any observed effects but rather to set limits on the sizes of the possible off-diagonal terms using current measurements. When these are as large as the diagonal terms, we are in the regime we have called MaxSFV.

This analysis builds upon that in the previous sections, in which we analysed the most powerful flavour constraints in representative no-scale SU(5) GUT scenarios. To this end, we consider scalar mass-squared matrices with universal flavour-diagonal entries and consider the effects of switching real off-diagonal real entries on, one at a time. There are in fact far too many possible parameter combinations in general. Thus, in order to see the effect of the additional (off-diagonal) parameters and to be concrete, we make some simplifying assumptions and test these one by one (in terms of generations).

Thus we consider matrices of the form

24 $$\begin{aligned} m^2_{\bar{5}}=\left( \begin{array}{lll} a &{} b &{} 0\\ b &{} a &{} 0\\ 0 &{} 0 &{} a \end{array} \right) , m^2_{\bar{5}}=\left( \begin{array}{lll} a &{} 0 &{} b\\ 0 &{} a &{} 0\\ b &{} 0 &{} a \end{array} \right) , m^2_{\bar{5}}=\left( \begin{array}{lll} a &{} 0 &{} 0\\ 0 &{} a &{} b\\ 0 &{} b &{} a \end{array} \right) , \end{aligned}$$

and

25 $$\begin{aligned} m^2_{10}=\left( \begin{array}{lll} c &{} d &{} 0\\ d &{} c &{} 0\\ 0 &{} 0 &{} c \end{array} \right) , m^2_{10}=\left( \begin{array}{lll} c &{} 0 &{} d\\ 0 &{} c &{} 0\\ d &{} 0 &{} c \end{array} \right) , m^2_{10}=\left( \begin{array}{lll} c &{} 0 &{} 0\\ 0 &{} c &{} d\\ 0 &{} d &{} c \end{array} \right) ,\nonumber \\ \end{aligned}$$

for $a\ne b$ and $c\ne d$, with all four parameters real. The rest of the boundary conditions at $M_{ in }$ are taken to be the same as in Sect. 2.2. We start by considering separately the boundary conditions (24) and (25), treating each sector separately.

**Fig. 10:**
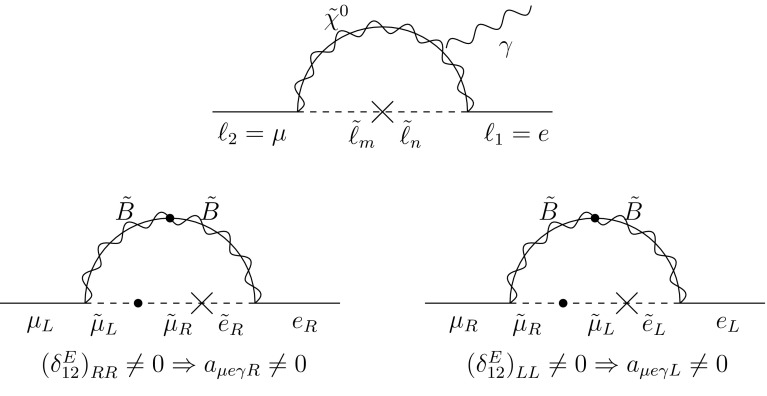
*Top* loop diagram representing the transition $\ell _2 \rightarrow \ell _1 \gamma$ in mass eigenstates, with $\ell _i$ being the lepton mass eigenstates, in our Ansatz 1 $\ell _2=\mu$ and $\ell _1=e$, and $\tilde{\ell }_m$ the sleptons eigenstates, $m=1,2,\ldots , 6$. *Bottom* leading contributions to $a_{\mu e \gamma }$ in the case of large $\mu$, represented in the flavour basis

### Off-diagonal entries in $\mathbf {m}^2_{\bar{\mathbf {5}}}$ and $\mathbf {m}^2_{{\mathbf {10}}}$

#### The (1, 2) sector

The inputs (24) imply the following universal matching conditions at $M_{ GUT }$:

26 $$\begin{aligned} m^2_{D _{12}}=m^2_{L _{12}}=m^2_{\bar{5}_{12}} \, , \end{aligned}$$

which will have the strongest impact on observables where $m^2_{D _{12}}$, $m^2_{L _{12}}$ enter at one loop level. In the lepton sector, this is the case for the amplitude of the process $\mu \rightarrow e \gamma$, shown in Fig. 10. This amplitude can be written as $\mathcal {M}_{\mu e \gamma }$ $= \frac{e}{2m_\mu }\, \epsilon ^{*\alpha } \bar{u}_e(k+q) \,[ i \sigma _{\beta \alpha } q^\beta ( a_{\mu e \gamma R} P_L + a_{\mu e \gamma L} P_R) ] \, u_\mu (k)$ where $\sigma _{\alpha \beta } = i/2 \, [\gamma _\alpha ,\gamma _\beta ]$, $\epsilon ^\alpha$ is the photon polarisation vector, *k* and $k+q$ are on-shell momenta, $u_\mu$, $\bar{u}_\mu$ are spinors that satisfy the Dirac equation and $P_{R,L}=(1\pm \gamma _5)/2$. The *L* / *R* index in $a_{\mu e \gamma L/R}$ refers to the electron chirality. In terms of $a_{\mu e \gamma L}$ and $a_{\mu e \gamma R}$, we have

27 $$\begin{aligned} \text {BR} (\mu \rightarrow e \gamma )= \frac{3\pi ^2 e^2}{G_F^2 m^4_\mu } \, ( |a_{\mu e \gamma L}|^2 + |a_{\mu e \gamma R}|^2 ). \end{aligned}$$

In fact, if $\tan \beta$ is large and $\mu>M_2>M_1$, the dominant contribution to $\text {BR} (\mu \rightarrow e \gamma )$ is mediated by bino exchange, which is represented in the flavour basis by the mass-insertion diagrams of the second row in Fig. 10. The contributions to $a_{\mu e \gamma L,R}$ in the mass eigenstate basis are schematically as follows [81]:

28 $$\begin{aligned} a_{\mu e \gamma L}\approx & {} g_1^2 \frac{m_\mu }{48\pi ^2} \sum _m K^*_{m1} K_{m5} \, \frac{M_1}{m^2_{L_m}} \, F_2^N\left( \frac{m^2_{\tilde{\chi }^0_1}}{m_{L_m}^2} \right) , \nonumber \\ a_{\mu e \gamma R}\approx & {} g_1^2 \frac{m_\mu }{48\pi ^2} \sum _m K^*_{m4} K_{m2} \, \frac{M_1}{m^2_{L_m}} \,F_2^N\left( \frac{m^2_{\tilde{\chi }^0_1}}{m_{L_m}^2} \right) , \end{aligned}$$

where the matrices *K* diagonalise the squared-mass matrices, (34). The mass eigenstate index 1 in $K^*_{m1}K_{m5}$, corresponds roughly to $\tilde{e}_L$ while the index 5 to $\tilde{\mu }_L$. Analogously in $K^*_{m4}K_{m2}$, the index 4 roughly corresponds to $\tilde{\mu }_R$, while the index 2 refers to $\tilde{e}_R$, and $F_2^N$ denotes the loop function involved in each diagram. All other contributions involve higgsinos and are therefore suppressed for large $\mu$. We note that in Eqs. (28), all indices $m=1,2,\ldots , 6$ give contributions to $\text {BR}(\mu \rightarrow e\gamma )$.

**Fig. 11:**
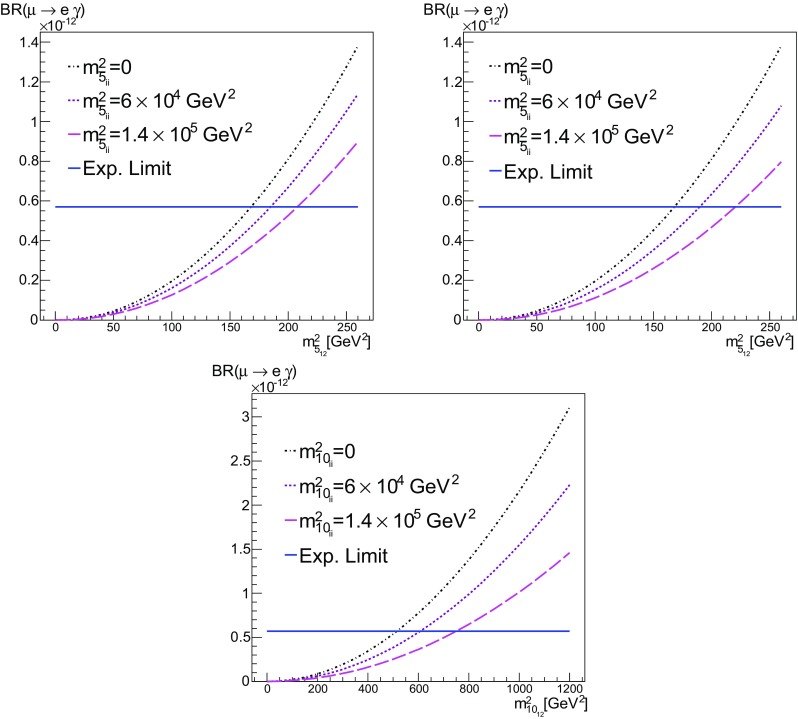
*Top* $b = m^2_{\bar{5}_{12}}$ vs. $\text {BR}(\mu \rightarrow e\gamma )$ for $a = m^2_{\bar{5}_{ii}}=0$, $6\times 10^4$ GeV$^2$ and $1.4\times 10^5$ GeV$^2$. In the *first panel*, $m^2_{10}=0$ ($c=d=0$), while in the second $m^2_{10_{ii}}=m^2_{\bar{5}_{ii}}$ ($c=a$, $d=0$). *Bottom* $d = m^2_{10_{12}}$ vs. $\text {BR}(\mu \rightarrow e\gamma )$, where only off-diagonal elements of $m^2_{10}$ were set using $m^2_{10_{ii}}=m^2_{\bar{5}_{ii}}$ (that is, $c=a$, $b=0$)

When $\hat{m}^2_{E (\mathrm RR)_{12}}$ is significantly suppressed with respect to $\hat{m}^2_{E (\mathrm LL)_{12}}$, which is our case since the seed for $\hat{m}^2_{E (\mathrm RR)}$ is $m^2_{10}$, whereas the seed for $\hat{m}^2_{E (\mathrm LL)}$ is $m^2_{5}$, then $a_{\mu e \gamma L}$ dominates. Its main contributions come from mass eigenstates mainly containing $\tilde{e}_L$, $\tilde{\mu }_L$ and $\tilde{\mu }_R$, provided that flavour-violating LR parameters are smaller than LL and RR, which is also our case. Then the most important contributions in $a_{\mu e \gamma L}$ come when $\tilde{\ell }_m=$ $\tilde{e}_L$, $\tilde{\mu }_L$ and $\tilde{\mu }_R$, and for each of them

29 $$\begin{aligned}&K^*_{m1}K_{m5}\approx \left. t_{\tilde{l}_m} (\delta ^E_{22})_\mathrm{LR} (\delta ^E_{12})_\mathrm{LL} \right| _{\tilde{\ell }_m\approx \tilde{e}_L, \tilde{\mu }_L, \tilde{\mu }_R}, \end{aligned}$$

where $$t_{\tilde{l}_m} $$ is a number of *O*(1) and is different for each of the terms above [81]. Hence, once $$m^2_{\bar{5}_{12}}$$ is non-zero at the input scale, the parameter $$m^2_{L _{12}}$$ is significantly bigger at the GUT scale than in the no-scale supergravity case, and this directly impacts the increase of $$a_{\mu e \gamma L}$$ and hence $$\text {BR}(\mu \rightarrow e\gamma )$$. In the quark sector, the analogous increase of $$ m^2_{D _{12}}$$ at the GUT scale will have an impact in the analogous amplitude to $$a_{\mu e \gamma R}$$, that is, $$a_{sd\gamma R}$$, but this time the Standard Model contribution to the decay $$s\rightarrow d \gamma $$ will be the dominant one, with supersymmetry making only a tiny correction [82]. We observe that $$m^2_{D _{12}}$$ affects the observable $$\text {BR}(B_d\rightarrow \mu ^+\mu ^-)$$ because it enters through a penguin diagram with higgsinos and sleptons in the loop, but also in this case the contribution is tiny in comparison to the SM contribution [83]. Finally, $$\Delta m_K$$ and hence $$\epsilon _K$$ are affected by $$m^2_{D _{12}}$$ but in this case the contributions coming from the small flavour-violating parameters in Ansatz 1 do not have an effect, as these parameters should typically be of $$O(10^{-2})$$ to have an impact in changing the values of $$\epsilon _K$$ and $$\Delta m_K$$ [79].

We plot in Fig. 11 the value of $\text {BR}(\mu \rightarrow e\gamma )$ as a function of $b = m^2_{\bar{5}_{12}}$, for the three choices $a = m^2_{\bar{5}_{ii}}=0$, $6\times 10^4$ GeV$^2$ and $1.4\times 10^5$ GeV$^2$. In the first panel we take $m^2_{10_{ii}}=0$ ($c=d=0$), whereas in the second panel $m^2_{10_{ii}}=m^2_{\bar{5}_{ii}}$ ($c=a$, $d=0$). The results are quite similar, though there are differences in the supersymmetric spectra. In both cases we find that $\text {BR}(\mu \rightarrow e\gamma )$ requires $m^2_{\bar{5}_{12}} = b \lesssim 170$ GeV$^2$ for small $m^2_{\bar{5}_{ii}} = a \rightarrow 0$. This means that flavour violation among the scalar masses-squared in the (1, 2) sector could be maximal, i.e., $b/a \sim 1$, if the universal diagonal entry $m^2_{\bar{5}_{ii}} = a \lesssim 170$ GeV$^2$. As one would expect, larger values of $m^2_{\bar{5}_{12}}$ would be allowed for larger values of $m^2_{\bar{5}_{ii}}$, but the ratio *b* / *a* could not reach unity. We consider the choice $m^2_{\bar{5}_{ii}}=1.4\times 10^5$ GeV$^2$ as an upper limit for *a* since, for higher values, the spectrum is sufficiently different from our original benchmark **B** that it no longer satisfies the constraint on the relic density. In this case, the upper limit on *b* is 210 (220) GeV$^2$ for $m^2_{10_{ii}}=0$ ($= a$), so we must require $b \ll a$. In the third panel of Fig. 11 we consider the effects of the off-diagonal components in $m^2_{10}$. Here, we have set $m^2_{10_{ii}}=m^2_{\bar{5}_{ii}}$ with $m^2_{\bar{5}_{12}} =0$ ($c=a$, $b=0$), and have plotted the branching ratio as a function of $d = m^2_{10_{12}}$. As one can see, the constraint on *d* is much weaker than the analogous constraint on *b*.

**Table 8:** Summary of constraints on $b = a$ and $d = c$ in the MaxSFV scenario. All quantities are in GeV$^2$.

Sector	(12)	(13)	(23)
$m^2_{\bar{5}}$ – present	170	8000	–
$m^2_{\bar{5}}$ – future	–	230	3400
$$ m^2_{10}$$ – present	520	1800	–
$$ m^2_{10}$$ – future	–	1550	73,000

**Fig. 12:**
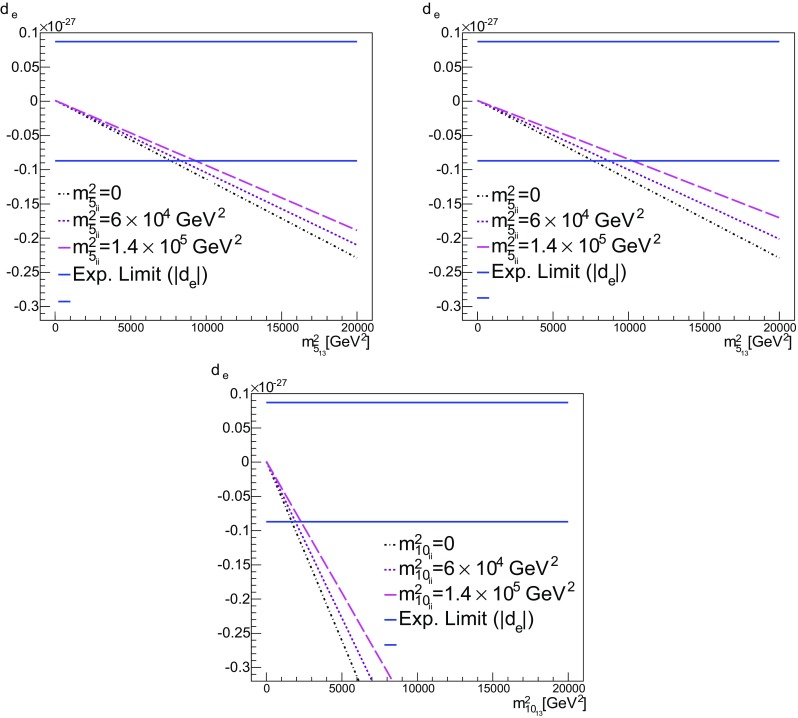
*Top* we show $b = m^2_{\bar{5}_{13}}$ vs. $d_e$ for $a = m^2_{\bar{5}_{ii}}=0$, $6\times 10^5$ GeV$^2$ and $1.4\times 10^5$ GeV$^2$; in the *first panel* $m^2_{10}=0$ ($c=d=0$), while in the *second panel* $m^2_{10_{ii}}=m^2_{5_{ii}}$ ($c=a$, $d=0$). *Bottom* here we show $d = m^2_{10_{13}}$ vs. $d_e$ for $m^2_{\bar{5}_{ii}}=m^2_{10_{ii}}$ ($c=a$, $b=0$)

We conclude that the MaxSFV scenario is possible in the (1, 2) sector if $m^2_{\bar{5}_{12}} = b \lesssim 170$ GeV$^2$, and if $m^2_{10_{12}} = d \lesssim 520$ GeV$^2$ as summarised in Table 8.

#### The (1, 3) sector 

We see from (31) that, once the Yukawa couplings are complex, the soft-squared masses and trilinear terms also become complex. We remind the reader that the only source of CP violation stems from the CKM phase, which through (15) translates into the complex Yukawa couplings, (14). Then, once an imaginary seed for $m^2_{\bar{5}_{13}}$ is set, a real non-zero entry at $M_{ in }$ for $m^2_{\bar{5}_{13}}$ will increase faster for both the imaginary and real parts of $m^2_{\bar{5}_{13}}$, than in the case of the pure no-scale set up.

**Fig. 13:**
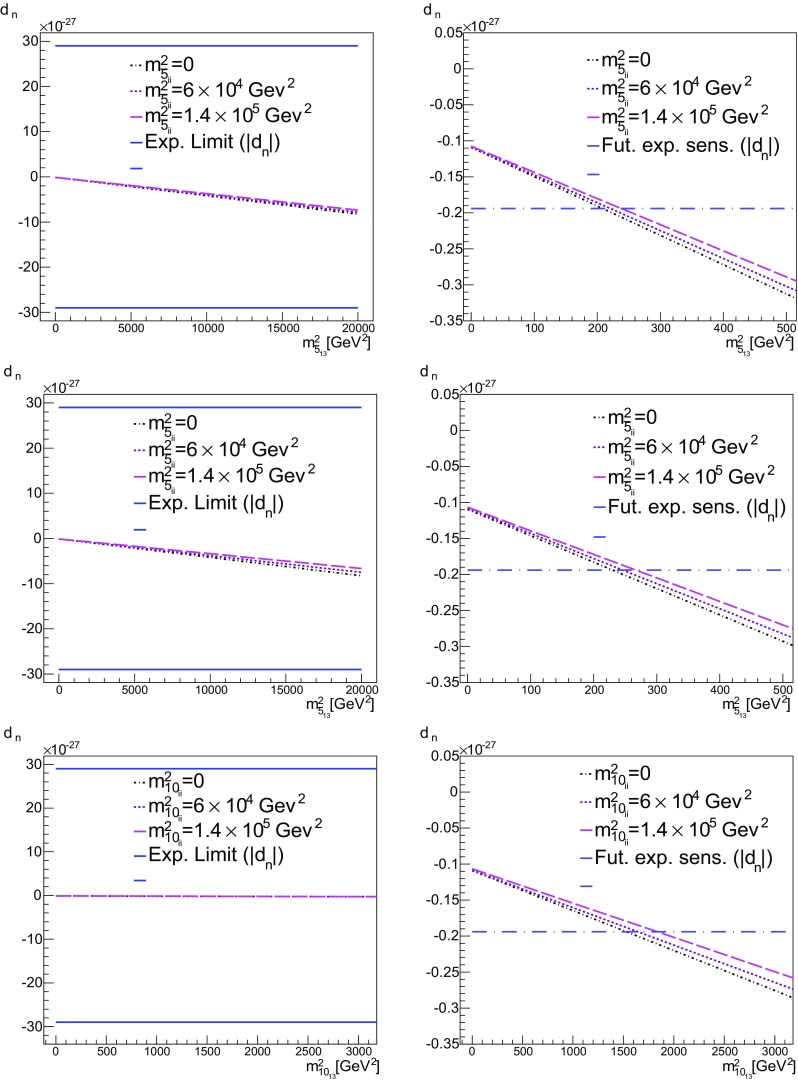
*Top* $b = m^2_{\bar{5}_{13}}$ vs. $d_n$ for $a = m^2_{\bar{5}_{ii}}=0$, $6\times 10^5$ GeV$^2$ and $1.4\times 10^5$ GeV$^2$. Here $m^2_{10}=0$ ($c=d=0$). For definiteness, we have used the value of $1.94\times 10^{-28}$ [84], for the future sensitivity. Other values (all of $O(10^{-28})$) can be found in [73]. *Middle* $m^2_{10_{ii}}=m^2_{5_{ii}}$ ($c=a$, $d=0$). *Bottom* $d = m^2_{10_{13}}$ vs. $d_n$ for $m^2_{10_{ii}}=m^2_{\bar{5}_{ii}}$ ($c=a$, $b=0$)

**Fig. 14:**
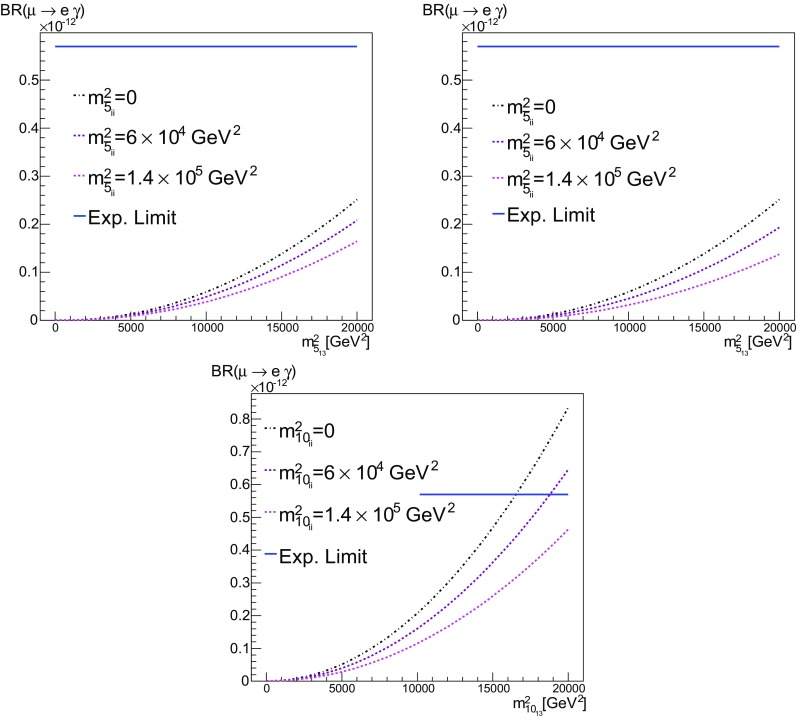
*Top* $b = m^2_{\bar{5}_{13}}$ vs. $a = \text {BR}(\mu \rightarrow e\gamma )$ for $m^2_{\bar{5}_{ii}}=0$, $6\times 10^4$ GeV$^2$ and $1.4\times 10^5$ GeV$^2$. In the *first* *panel* $m^2_{10}=0$ ($c=d=0$), while in the *second* *panel* $m^2_{10_{ii}}=m^2_{5_{ii}}$ ($c=a$, $d=0$). *Bottom* $d = m^2_{10_{13}}$ vs. $\text {BR}(\mu \rightarrow e\gamma )$ for $m^2_{10_{ii}}=m^2_{5_{ii}}$ ($c=a$, $b=0$)

We see from Table 6 that in A1 the value of the electron EDM, $d_e$, is already close to the experimental limit and from the diagram in Fig. 9 we see the potential importance for this observable when increasing the real part of $m^2_{\bar{5}_{13}}$. In A1, the leading contribution to $d_e$ comes from the terms in (24) and once ${\mathrm{{Re}}}[m^2_{\bar{5}_{13}}]$ is non-zero at $M_{ in }$, then at the GUT scale it will be bigger than in the no-scale case and particularly ${\mathrm{{Re}}}[(\delta ^D_{31})_\mathrm{RR}]$ will have a significant increase. As a consequence, for ${\mathrm{{Re}}}[m^2_{\bar{5}_{13}}] \ne 0$ at the input scale, the most constraining observable is the electron EDM. Figure 12 shows $d_e$ as a function of $b = m^2_{\bar{5}_{13}}$. The bounds on the electron EDM are shown by the two horizontal solid lines straddling $d_e = 0$. As one can see, the flavour off-diagonal entry in the squark mass matrix is bounded by $b < 10^4$ GeV$^2$ for our maximal value of *a*. Once again, allowing $c = a$ (with $d = 0$) does not greatly affect this limit. On the other hand, when we consider the EDM as a function of $d = m^2_{10_{13}}$ (with $b = 0$), we find that $d < 2000$ GeV$^2$ for the maximal value of $c = a$.

The neutron EDM is well below the present experimental limit, but at close to the future sensitivity of $O(10^{-28})$ e cm [73]. In Fig. 13 we show the neutron EDM as a function of $m^2_{\bar{5}_{13}}$ for three values of $m^2_{\bar{5}_{ii}}=0$, $6\times 10^4$ GeV$^2$ and $1.4\times 10^5$ GeV$^2$. The left panels shows the current (lack of) constraints, while the right panels show the anticipated future constraints. In the top row, $m^2_{10}=0$ ($c = d = 0$). In the middle row, $m^2_{10_{ii}}=m^2_{5_{ii}}$ ($c=a$, $d=0$), and in the bottom row, we plot $d_n$ versus $d = m^2_{10_{13}}$ with $c = a$ and $b = 0$. As one can see, there are no current constraints on either *b* or *d* for our allowed range in *a* and *c*. However, we expect that future constraints can place a limit of about $b \lesssim 230$ GeV$^2$ and $d \lesssim 1540$ GeV$^2$.

Other parameters that are affected by switching on a non-zero off-diagonal parameter are $\epsilon _K$, $\text {BR}(\mu \rightarrow e\gamma )$ and $\text {BR}(\tau \rightarrow e\gamma )$, which remain (for the most part) within the experimental limits. Figure 14 shows the case for $\text {BR}(\mu \rightarrow e\gamma )$. This branching ratio is particularly sensitive to the increase in $d = m^2_{10_{13}}$ if this is allowed to grow much faster than $m^2_{10_{12}}$ and $m^2_{\bar{5}_{12}}$, which is depicted in the third panel in Fig. 14. When the real part of $m^2_{10_{13}}$ is non-zero at $M_{ in }$, while the real parts of $m^2_{10_{12}}$ and $m^2_{\bar{5}_{12}}$ are zero, the values of $m^2_{E_{12}}$ and $m^2_{L_{12}}$ will evolve to values close to their no-scale values at $M_{ EW }$, producing a value for $a_{\mu e \gamma L}$ close to the no-scale case. When $m^2_{10_{13}}$ is allowed to increase to $O(10^4)$ GeV$^2$, the $a_{\mu e \gamma R}$ contribution to $\text {BR}(\mu \rightarrow e\gamma )$, will dominate over $a_{\mu e \gamma L}$, and the terms in $a_{\mu e \gamma R}$, (28), for $m=6$, which corresponds to the lightest slepton, will drive $\text {BR}(\mu \rightarrow e\gamma )$ to levels above the experimental bound.

**Fig. 15:**
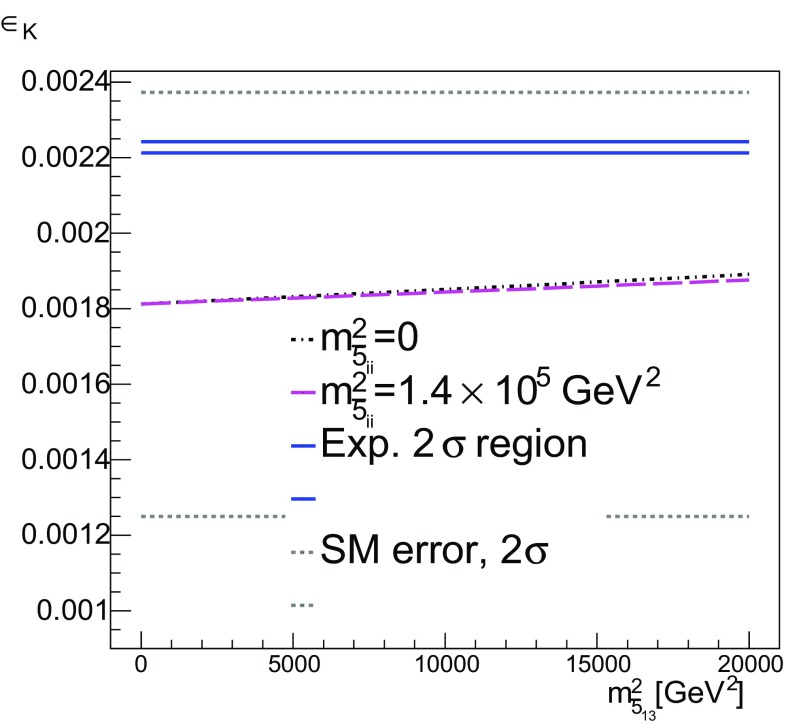
The 2 $\sigma$ experimental region of $|\epsilon _K|$ is (2.213 –$2.243)\times 10^{-3}$, and the range of the supersymmetric prediction together with the 2 $\sigma$ Standard Model error is (1.25 –$2.37)\times 10^{-3}$

In the case of $\epsilon _K$, the Standard Model uncertainty, which must be added to the supersymmetric value, must be taken into account. In Fig. 15 we show $\epsilon _K$ as a function of $b = m^2_{\bar{5}_{13}}$ together with the area allowed by the NNLO SM error [80] and the 2 $\sigma$ experimental region. This clarifies that the value that we obtain for $\epsilon _K$ is in agreement with observations.

#### The (2, 3) sector

**Fig. 16:**
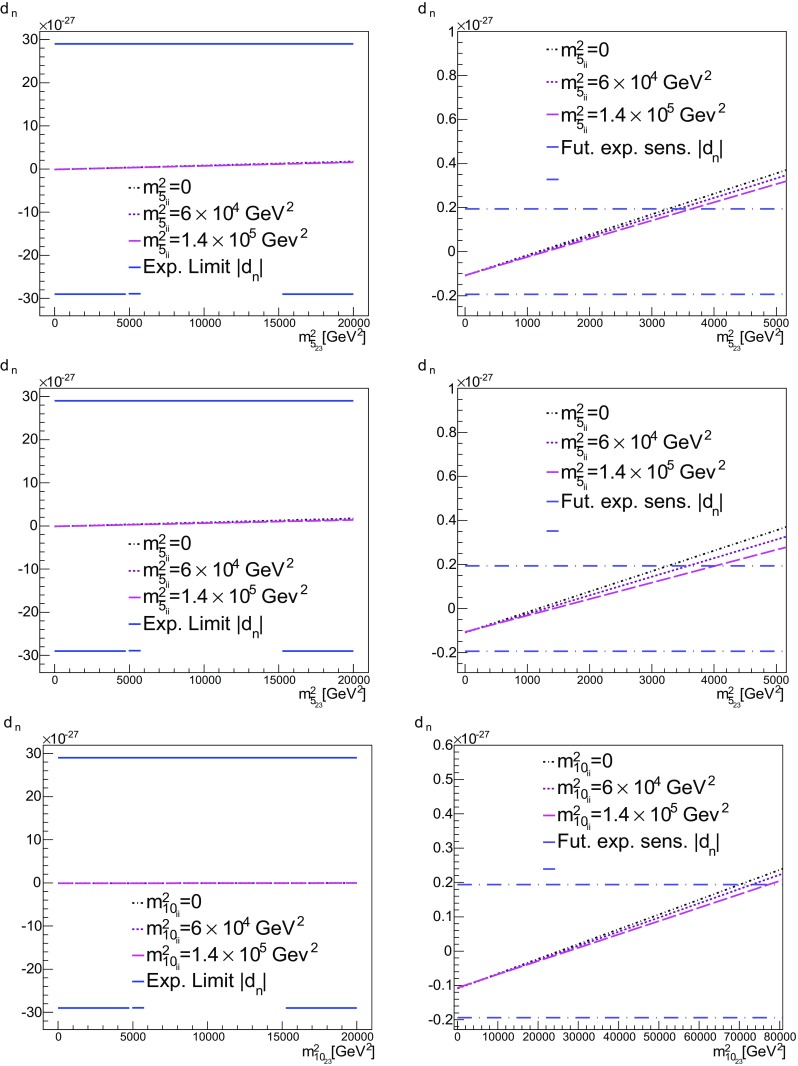
*Top* $m^2_{\bar{5}_{23}}$ vs. $d_n$ for $m^2_{\bar{5}_{ii}}=0$, $6\times 10^5$ GeV$^2$ and $1.4\times 10^5$ GeV$^2$. Here $m^2_{10_{ii}}=0$ ($c=d=0$). For definiteness, we have used the value of $1.94\times 10^{-28}$ [84], for the future sensitivity. Other values (all of $O(10^{-28})$) can be found in [73]. *Middle* the same as above, except for $m^2_{10_{ii}}=m^2_{5_{ii}}$ ($c=a$, $d=0$). *Bottom* the case for $m^2_{10_{23}}$ vs. $d_n$ for $m^2_{10_{ii}}=m^2_{\bar{5}_{ii}}$ ($c=a$, $b=0$)

In this sector the most constraining parameter is the neutron EDM. Just as in the case of sector (1, 3), the neutron EDM is well below the present experimental limit and up to a value of $m^2_{\bar{5}_{23}}\approx 3.2\times 10^3$ GeV$^2$ (for $m^2_{\bar{5}_{ii}}=0$) also below the expected limit of the future sensitivity of $O(10^{-28})$  e cm. In Fig. 16 we show the neutron EDM as a function of $m^2_{\bar{5}_{23}}$ for three values of $m^2_{\bar{5}_{ii}}=0$, $6\times 10^4$ GeV$^2$ and $1.4\times 10^5$ GeV$^2$. As in Fig. 13, we show current constraints from the neutron EDM in the left panels and future limits on right. In the top row for the present experimental limit, while in the second for the future sensitivity. In the top row, $m^2_{10}=0$ ($c = d = 0$). In the middle row, $m^2_{10_{ii}}=m^2_{5_{ii}}$ ($c=a$, $d=0$), and in the bottom row, we plot $d_n$ versus $d = m^2_{10_{23}}$ with $c = a$ and $b = 0$. As one can see, there are no current constraints on either *b* or *d* for our allowed range in *a* and *c*. However, we expect that future constraints can place a limit of about $b \lesssim 3500$ GeV$^2$ and $d \lesssim 8\times 10^4$ GeV$^2$.

Other parameters that are affected by allowing for a non-zero off-diagonal parameter in the (23) sector are $\epsilon _K$, $\text {BR}(\mu \rightarrow e\gamma )$, $\text {BR}(\tau \rightarrow \mu \gamma )$ and $\text {BR}(\tau \rightarrow e\gamma )$, which, however, remain within the experimental limits. Once again, for $\epsilon _K$ we also rely on taking into account the Standard Model uncertainty to obtain compatibility. We note also that $\text {BR}(B\rightarrow X_s\gamma )$ is sensitive to changes in this sector, though this is noticeable only for light ($\lesssim$1 TeV) supersymmetric spectra [51].

Table 8 summarise the constraints on the MaxSFV scenario in the (12), (13) and (23) sectors. The numbers in the various entries of the table are the maximum values in GeV$^2$ of *b* (in the soft supersymmetry-breaking fiveplet mass-sequared matrix) where $b/a = 1$ is allowed by present (and possible future) data, and the maximum values of *b* (in the soft supersymmetry-breaking tenplet mass-sequared matrix) where $d/c = 1$ is allowed by present (and possible future) data. We see that the MaxSFV scenario in the fiveplet (12) sector is consistent with the present data for $m^2_{\bar{5}} \lesssim 170$ GeV$^2$, increasing to $\lesssim$520 GeV$^2$ in the tenplet (12) sector, $\lesssim$8000 GeV$^2$ in the fiveplet (13) sector, and $\lesssim$1800 GeV$^2$ in the tenplet (13) sector. There are currently no constraints on MaxSFV in the (23) sector, though this may change with future data.

## Summary

We have explored in this paper the phenomenological constraints on super-GUT models, in which soft supersymmetry-breaking inputs are postulated at some scale $M_{ in }$ intermediate between $M_{ GUT }$ and the Planck scale, that are imposed by upper limits on flavour and CP violation. For this purpose, we have chosen a benchmark supersymmetric model (**B** in (19) above) motivated by no-scale supergravity that is consistent with other constraints from the LHC (e.g., $m_h$ and the non-appearance of sparticles during Run 1) and cosmology (e.g., the density of cold dark matter and inflation). Within this framework we have considered four possible scenarios for Yukawa couplings that are compatible with CKM mixing and its extension to sparticles. Consideration of the runnings of model parameters in two of these scenarios (A3 and A4 in Eqs. (17) and (18) above) were found to be generally *incompatible* with the flavour-violation constraints, and not pursued further. However, the other two scenarios (A1 and A2 in Eqs. (15) and (16) above) were found to be *compatible* with the flavour-violation constraints. They have quite different predictions, and they serve to illustrate the range of possibilities for future flavour-violation measurements in no-scale super-GUTs.

We then considered possible deformations of the no-scale scenario, in which the soft supersymmetry-breaking scalar masses $m_0$ are allowed to be non-zero but much smaller than the gaugino mass $m_{1/2}$ at $M_{ in }$. In particular, we have investigated the maximal magnitudes of off-diagonal terms in the sfermion mass-squared matrices $m_0^2$ for the SU(5) fiveplets and tenplets, and the possibility that these might be as large as the diagonal entries, a scenario we call MaxSFV. We find that the off-diagonal (12) entry in the fiveplet mass-squared matrix could be as large as the diagonal entries if the latter are $\simeq$170 GeV$^2$ and $\simeq$520 GeV$^2$ in the $m^2_{\bar{5}}$ and $m^2_{10}$ mass-squared matrices, respectively. The corresponding numbers in the (13) sector are $\simeq 8\times 10^3$ GeV$^2$ in the fiveplet mass-squared matrix and $\simeq 1.8\times 10^3$ GeV$^2$ for the tenplets. There are currently no useful bounds in the (23) sector. The future sensitivity in $d_n$ would be sensitive to MaxSFV in the (13) sector of $\mathcal{O}(230)$ GeV$^2$ for the for the fiveplets and of $\mathcal{O}(1.5\times 10^3)$ GeV$^2$ for the tenplets, and of $\mathcal{O}(3.4\times 10^3)$ GeV$^2$ in the (23) sector for the fiveplets and $\mathcal{O}(7.3\times 10^4)$ GeV$^2$ for the tenplets.

Within these limits, *there would be no supersymmetric flavour problem* associated with sfermion masses in the class of near-no-scale super-GUTs discussed in this paper.

## Appendix A: Three-family beta functions in SU(5)

Many of the RGEs for the single-family case can be found in [34, 40, 42, 43, 85]. Here we list the complete set of RGEs relevant for the 3-family case. Whilst the beta function for $\lambda '$ in EQNO does not change with respect to the one-family case, that is,

$$\begin{aligned} \frac{\text {d}\lambda '}{\text {d}t} = \frac{\lambda '}{16 \pi ^2} \left( \frac{63 \lambda '^2}{20}+3 \lambda ^2 \ - 30 {g}_{5}^2\right) , \nonumber \end{aligned}$$

the other beta functions change as follows:

$$\begin{aligned} \frac{\text {d}\lambda }{\text {d}t}= & {} \frac{\lambda }{16 \pi ^2} \left( 48 \text {Tr}[{h}_{10}^ {\dagger }{h}_{10}]+2 \ \text {Tr}[{h}_{\bar{5}}^ {\dagger }{h}_{\bar{5}}] \right. \nonumber \\&+\left. \frac{53 \lambda ^2}{5}+\frac{21 \lambda '^2}{20}-\frac{98 \ {g}_{5}^2}{5}\right) , \nonumber \\ \frac{\text {d}h_{10}}{\text {d}t}= & {} \frac{1}{16 \pi ^2} \left[ \ {h}_{10} \left( 48 \text {Tr}[{h}_{10}^ {\dagger }{h}_{10}]+\frac{24 \ \lambda ^2}{5}-\frac{96 {g}_{5}^2}{5}\right) \right. \nonumber \\&+\,96 {h}_{10}{h}_{10}^ {\dagger }{h}_{10} + \left. {h}_{10}{h}_{\bar{5}}^ {\dagger }{h}_{\bar{5}}+{h}_{\bar{5}} {h}_{\bar{5}}^ {\dagger }{h}_{10} \right] , \nonumber \\ \frac{\text {d}h_{\bar{5}}}{\text {d}t}= & {} \frac{1 }{16 \pi ^2} \left[ {h}_{\bar{5}} \left( 2 \text {Tr}[{h}_{\bar{5}}^ {\dagger }{h}_{\bar{5}}]+\frac{24 \lambda ^2}{5}-\frac{84 \ {g}_{5}^2}{5}\right) \right. \nonumber \\&+ \, \left. +48 {h}_{\bar{5}}{h}_{10}^ {\dagger }{h}_{10}3 \ {h}_{\bar{5}} {h}_{\bar{5}}^ {\dagger } {h}_{\bar{5}} \right] ; \nonumber \end{aligned}$$

30 $$\begin{aligned} \frac{\text {d}A_\lambda }{\text {d}t}= & {} \frac{1}{8 \pi ^2} \left[ 48 \text {Tr}[{h}_{10}^ {\dagger }{a}_{10}]+2 \ \text {Tr}[{h}_{\bar{5}}^ {\dagger }{a}_{\bar{5}}] \right. \nonumber \\&\left. +\frac{53 \lambda ^2 {A}_{\lambda }}{5}+\frac{21 \ {A}_{\lambda '} \lambda '^2}{20} - \frac{98 {g}_{5}^2 {M}_{5}}{5}\right] , \nonumber \\ \frac{\text {d}A_{\lambda '}}{\text {d}t}= & {} \frac{1}{8 \pi ^2} \left[ 3 \lambda ^2 {A}_{\lambda }+\frac{63 {A}_{\lambda '} \lambda '^2}{20}-30 {g}_{5}^2 \ {M}_{5}\right] , \nonumber \\ \frac{\text {d}a_{10}}{\text {d}t}= & {} \frac{1}{16 \pi ^2}\left[ \frac{24}{5} {a}_{10} \left( 10 \text {Tr}[{h}_{10}^\dagger {h}_{10}]+\lambda ^2-8 {g}_{5}^2\right) \right. \nonumber \\&+ \, \frac{48}{5} {h}_{10} \left( 10 \text {Tr}[{h}_{10}^\dagger {a}_{10}]+ |\lambda |^2 {A}_{\lambda } -4 {g}_{5}^2 {M}_{5}\right) \nonumber \\&+ \, 144 {a}_{10} {h}_{10}^\dagger {h}_{10} + 144 \ {h}_{10} {h}_{10}^\dagger {a}_{10} +{a}_{10} {h}_{\bar{5}}^\dagger {h}_{\bar{5}}\nonumber \\&\, \left. +{h}_{\bar{5}} {h}_{\bar{5}}^\dagger {a}_{10} +2 ({a}_{\bar{5}} {h}_{\bar{5}}^\dagger {h}_{10}+{h}_{10} {h}_{\bar{5}}^\dagger {a}_{\bar{5}}) \right] ,\\ \frac{\text {d}a_{\bar{5}}}{\text {d}t}= & {} \frac{1}{16 \pi ^2} \left[ {h}_{\bar{5}} \left( 4 \text {Tr}[{h}_{\bar{5}}^ {\dagger }{a}_{\bar{5}}]+\frac{48 \lambda ^2 \ {A}_{\lambda }}{5}- \frac{168 {g}_{5}^2 {M}_5 }{5}\right) \right. \nonumber \\&+ \, {a}_{\bar{5}} \left( 2 \ \text {Tr}[{h}_{\bar{5}}^ {\dagger }{h}_{\bar{5}}]+\frac{24 |\lambda |^2}{5} -\frac{84 \ {g}_{5}^2}{5}\right) \nonumber \\&+96 {h}_{\bar{5}}{h}_{10}^ {\dagger }{a}_{10} + \, 48 \ {a}_{\bar{5}}{h}_{10}^ {\dagger }{h}_{10} \nonumber \\&\left. +6 {a}_{\bar{5}}{h}_{\bar{5}}^ {\dagger }{h}_{\bar{5}}\ + 3 {h}_{\bar{5}}{h}_{\bar{5}}^ {\dagger }{a}_{\bar{5}} \right] ; \nonumber \end{aligned}$$

31 $$\begin{aligned} \frac{\text {d}m_{24}^{2}}{\text {d}t}= & {} \frac{1}{8 \pi ^2} \left[ \lambda ^2 \ \left( {A}_{\lambda }^2+{m}_{24}^{2}+{m}_{5_H}^{2}+{m}_{\bar{5}_H}^{2}\right) \right. \nonumber \\&\left. +\frac{21}{20} \ \lambda '^2 \left( {A}_{\lambda '}^2+3 {m}_{24}^{2}\right) -20 {g}_{5}^2 {M}_{5}^2 {\mathbf {1}} \right] \, , \nonumber \\ \frac{\text {d}m_{5_H}^{2}}{\text {d}t}= & {} \frac{1}{8 \pi ^2} \left[ 3 (16 \text {Tr}[{a}_{10}^{\dagger }{a}_{10}]+32 \ \text {Tr}[{h}_{10}{m}_{10}^{2}{h}_{10}^ {\dagger }] \right. \nonumber \\&\left. +16 {m}_{5_H}^{2} \ \text {Tr}[{h}_{10}^ {\dagger }{h}_{10}])\right. \nonumber \\&+ \left. \frac{24}{5} \lambda ^2 \ \left( {A}_{\lambda }^2+{m}_{24}^{2}+{m}_{5_H}^{2}+{m}_{\bar{5}_H}^{2}\right) \right. \nonumber \\&-\left. \frac{48 {g}_{5}^2 \ {M}_{5}^2}{5} {\mathbf {1}} \right] \, , \nonumber \\ \frac{\text {d}m_{\bar{5}_H}^{2}}{\text {d}t}= & {} \frac{1}{8 \pi ^2} \left[ \frac{1}{2} 2 \ (\text {Tr}[{a}_{\bar{5}}^{\dagger }{a}_{\bar{5}}]+\text {Tr}[{h}_{\bar{5}}{m}_{10}^{2}{h}_{\bar{5}}^ {\dagger }]\ \right. \nonumber \\&+\text {Tr}[{h}_{\bar{5}}^ {\dagger }{m}_{\bar{5}}^{2}{h}_{\bar{5}} {\mathbf {1}} ] + \left. {m}_{\bar{5}_H}^{2} \ \text {Tr}[{h}_{\bar{5}}^ {\dagger }{h}_{\bar{5}}])+\frac{24}{5} \lambda ^2 \ \right. \nonumber \\&\left( {A}^2_{\lambda }+{m}_{24}^{2}+{m}_{5_H}^{2}+{m}_{\bar{5}_H}^{2}\right) - \left. \frac{48}{5} {g}_{5}^2 \ {M}_{5}^2 {\mathbf {1}} \right] \, , \nonumber \\ \frac{\text {d}m_{\bar{5}}^{2}}{\text {d}t}= & {} \frac{1}{16 \pi ^2} \left[ 2 (2 {a}_{\bar{5}}^{\dagger }{a}_{\bar{5}}+2 {m}_{\bar{5}_H}^{2} \ {h}_{\bar{5}}^ {\dagger }{h}_{\bar{5}}+{m}_{\bar{5}}^{2}{h}_{\bar{5}}^ {\dagger }{h}_{\bar{5}}\right. \nonumber \\&+ \left. 2 \ {h}_{\bar{5}}^ {\dagger }{m}_{10}^{2}{h}_{\bar{5}}+{h}_{\bar{5}}^ {\dagger }{h}_{\bar{5}}{m}_{\bar{5}}^{2}) -\frac{96 \ {g}_{5}^2 {M}_{{5}}^2}{5} {\mathbf {1}} \right] \, , \nonumber \\ \frac{\text {d}m_{10}^{2}}{\text {d}t}= & {} \frac{1}{16 \pi ^2} \left[ -\frac{144}{5} {g}_{5}^2 {M}_{5}^2 {\mathbf {1}} +{m}_{10}^{2}{h}_{\bar{5}}^ {\dagger }{h}_{\bar{5}}\right. \nonumber \\&+ \left. 96 ({m}_{5_H}^{2} {h}_{10}^ {\dagger }{h}_{10}+{h}_{10}^ {\dagger }{m}_{10}^{2}{h}_{10})\right. \nonumber \\&\left. + 48 \ ({m}_{10}^{2}{h}_{10}^ {\dagger }{h}_{10}+{h}_{10}^ {\dagger }{h}_{10}{m}_{10}^{2})\right. \nonumber \\&+ \left. 2 (48 {a}_{10}^{\dagger }{a}_{10}+{a}_{\bar{5}}^{\dagger }{a}_{\bar{5}}+{m}_{\bar{5}_H}^{2} \ {h}_{\bar{5}}^ {\dagger }{h}_{\bar{5}}+{h}_{\bar{5}}^ {\dagger }{m}_{\bar{5}}^{2}{h}_{\bar{5}}) \right. \nonumber \\&+ \left. {h}_{\bar{5}}^ {\dagger }{h}_{\bar{5}}\ {m}_{10}^{2} \right] \, ; \end{aligned}$$

32 $$\begin{aligned} \frac{\text {d}B_{24}}{\text {d}t}= & {} \frac{1}{8 \pi ^2} \left[ 2 \lambda ^2 {A}_{\lambda }+\frac{21 {A}_{\lambda '} \lambda '^2}{10} -20 {g}_{5}^2 {M}_{5} {\mathbf {1}} \right] \, , \nonumber \\ \frac{\text {d}B_{5}}{\text {d}t}= & {} \frac{1}{8 \pi ^2} \left[ 48 \text {Tr}[{h}_{10}^ {\dagger }{a}_{10}]+2 \ \text {Tr}[{h}_{\bar{5}}^ {\dagger }{a}_{\bar{5}}]\right. \nonumber \\&+ \left. \frac{48 \lambda ^2 {A}_{\lambda }}{5} -\frac{48 \ {g}_{5}^2 {M}_{5}}{5} {\mathbf {1}} \right] \, . \end{aligned}$$

## Appendix B: Transformation rules

The physical mass eigenstates for each flavour are calculated in the SCKM basis by diagonalising the $6\times 6$ matrices

33 $$\begin{aligned} ({\mathcal {M}}^\mathrm{{SCKM}}_{ f})^2_{ij}= & {} \left[ \begin{array}{cc} \hat{m}^2_{ f \mathrm{LL}} &{} \hat{m}^2_{ f \mathrm{LR}}\\ \hat{m}^2_{ f \mathrm{LR}} &{} \hat{m}^2_{ f \mathrm{RR}} \end{array} \right] _{ij} \equiv (\widehat{\mathcal {M}}^{2}_{ f})_{ij} \nonumber \\= & {} \left[ \begin{array}{ll} &{}\left( V^{f\dagger }_{L}m^2_{ Q}V^{f}_L\right) _{ij}+(\hat{m}_{f_i})^2 \delta _{ij} +D^f_{L}\\ &{}-( (V^{f}_L a^{\dagger }_f V^{f\dagger }_{R})_{ij} v_f+\mu \tan ^p \beta \ \hat{m}_{f_i}\delta _{ij}) \end{array}\right. \nonumber \\&\left. \begin{array}{ll} &{}-((V^{f\dagger }_L a_{f} V^{f}_R)_{ij} v_f+\mu ^* \tan ^p\beta \ \hat{m}_{f_i}\delta _{ij} )\\ &{} (V^{f\dagger }_{R} m^2_{ f_R} V^{f}_R)_{ij}+ (\hat{m}_{f_i})^2\delta _{ij} + D^f_{R} \end{array}\right] \quad ,\nonumber \\ p= & {} \ \left\{ \begin{array}{c} 1,\ f=D, L\\ -1,\ f=U. \end{array}\right. , \end{aligned}$$

where $\hat{m}_{f}$ are the diagonal quark mass matrices. We use the following notation for the matrices diagonalising the soft mass-squared matrices:

34 $$\begin{aligned} K ({\mathcal {M}}^\mathrm{{SCKM}}_{ f})^2\ K^{\dagger } = \mathrm{{diag}}(m_{\tilde{\ell }_1}^2,\ldots ,m_{\tilde{\ell }_6}^2), \end{aligned}$$

where $m_{\tilde{\ell }_{i}}^{2}$ are the mass eigenstates.

To help understand the impact of the RGEs on the final parameter values at $M_\mathrm{EW}$, we show contributions to the beta functions in the SCKM basis taken from Eq. (4.34) of [86]:

35

where $m^2$ is a mass-squared term and not a matrix, for example $m^2=m^2_{H_d}$. Using $\mathrm{d}m^2=\int \mathrm{d}t \beta ^{(1)}_{m^2} / 16\pi ^2$, we can calculate the form of the mass-squared terms at the EW scale in the SCKM basis.

In addition to the transformation given in (20), the general transformations for all the terms appearing in (35), independent of the Ansatz, are as follows:

36 $$\begin{aligned}&{ {Transformation ~of ~terms ~in~ }} m^2_{ Q} {to ~the ~\mathrm{SCKM} ~basis:}\qquad {Transformation ~of ~terms ~in~ } m^2_{D}: \nonumber \\ 1.&m^2_{ Q}h_D^\dagger h_D \rightarrow \hat{m}^2_{ Q}\hat{h}_D^2 \qquad \qquad \qquad \qquad \qquad \qquad \qquad \qquad \qquad 1. ~ m^2_{D}h_D h_D^\dagger \rightarrow V^{d\dagger }_R m^2_{D}V^D_R \hat{h}^2_D \nonumber \\ 2.&m^2_{ Q}h_U^\dagger h_U \rightarrow \hat{m}^2_{ Q}V_{ CKM }^\dagger \hat{h}_U^2 V_{ CKM }\qquad \qquad \qquad \qquad \qquad \qquad 2.~h_D m^2_{ Q}h_D^\dagger \rightarrow \hat{h}_D \hat{m}^2_{ Q}\hat{h}_D \nonumber \\ 3.&~ h_U^\dagger h_U m^2_{ Q}\rightarrow V_{ CKM }^\dagger \hat{h}^2_U V_{ CKM }\hat{m}^2_{ Q}\qquad \qquad \qquad \qquad \qquad \qquad 3. ~ h_D h_D^\dagger m^2_{D}\rightarrow \hat{h}_D \left( V^{\dagger }_{DR} m^2_{D}V_{DR} \right) \nonumber \\ 4.&~ h_D^\dagger h_D m^2_{ Q}\rightarrow \hat{h}^2_D \hat{m}^2_{ Q}\qquad \qquad \qquad \qquad \qquad \qquad \qquad \qquad \qquad 4. ~ m^2 h_D h_D^\dagger \rightarrow m^2 \hat{h}_D \hat{h}^\dagger _D \nonumber \\ 5.&~ h_U^\dagger m^2_{U}h_U \rightarrow {V_{ CKM }}^\dagger \hat{h}_U \left( V^{u\dagger }_R m^2_{U}V^{U}_R \right) \hat{h}_U V_{ CKM }\nonumber \\ 6.&~ h_D^\dagger m^2_{D}h_D \rightarrow \hat{h}_D^\dagger \hat{m}^2_{D}\hat{h}_D \nonumber \\ 7.&~ a_D^\dagger a_D \rightarrow \hat{a}_D^\dagger \hat{a}_D&\nonumber \\ 8.&~ m^2 h_U^\dagger h_U \rightarrow m^2 V_{ CKM }^\dagger \hat{h}_U V_{ CKM }\nonumber \\ 9.&~ m^2 h_D^\dagger h_D \rightarrow \hat{h}^\dagger _D\hat{h}_D. \end{aligned}$$

The transformations (36) to the SCKM basis are valid at all energy scales. With the exception of the fifth entry in (36) for the transformations of $m^2_{ Q}$, the rest of the terms have the same form. (Actually, the initial value of $\hat{m}^2_{ Q}=V^{D\dagger }_L m^2_{ Q}V^D_L$ also has the same form, since $V^D_L=V_{ CKM }$ for both cases.) The fifth entry in (36) for the transformation of $m^2_{ Q}$, could potentially be different, because it is not given only in terms of $V_{ CKM }$ or squared quark masses, but $V^U_R$. However, since in both Ansätze we have the same form of $V^U_R$ and $m^2_{U}$, i.e., diagonal matrices, this term would not make a numerical difference in either Ansatz. Hence the only differences in $\hat{m}^2_{ Q}$ at $M_{ GUT }$ are the different values of the off-diagonal elements in $m^2_{ Q}$ and $m^2_{D}$. There are more differences in the transformations of terms in $\hat{m}^2_{D}$, since terms (1) and (3) of (36) explicitly involve $V^D_R$, which is different in the two cases.

37 $$\begin{aligned}&{ {Transformation ~of ~terms ~in~ }} a_D&\nonumber \\ 1.&~a_D h_D^\dagger h_D \rightarrow \hat{a}_{D}\hat{h}^2_D= & {} \, V^{D\dagger }_L a_D V^{D}_R\hat{h}^2_D&\nonumber \\ 2.&~a_D h_U^\dagger h_U \rightarrow \hat{a}_{D}\hat{h}^2_U V^{U\dagger }_R V^D_R= & {} \, V^{D\dagger }_L a_D V^{D}_R\hat{h}^2_U V^{U\dagger }_R V^D_R&\nonumber \\ 3.&~h_D h_U^\dagger a_D \rightarrow \hat{h}^2_D\hat{a}_{D}= & {} \, \hat{h}^2_DV^{D\dagger }_L a_D V^{D}_R&\nonumber \\ 4.&~h_D h_U^\dagger a_U \rightarrow \hat{h}_D \left( V^{D\dagger }_R V^U_R \hat{h}_U V^{U\dagger }_L a_U V^D_R \right) .&&\end{aligned}$$

## Footnotes

1 It is determined from $\mu _H$, $\lambda$, and the vacuum expectation value of $\Sigma$ [45].

2 Such assumptions can have important effects on the observables, and can help to determine the choices of Yukawa structures compatible with a given supersymmetric model [51].
